# Processing Complex Sounds Passing through the Rostral Brainstem: The New Early Filter Model

**DOI:** 10.3389/fnins.2016.00136

**Published:** 2016-05-10

**Authors:** John E. Marsh, Tom A. Campbell

**Affiliations:** ^1^School of Psychology, University of Central LancashirePreston, UK; ^2^Department of Building, Energy and Environmental Engineering, University of GävleGävle, Sweden; ^3^Neuroscience Center, University of HelsinkiHelsinki, Finland

**Keywords:** auditory brainstem response (ABR), complex auditory brainstem response (cABR), electroencephalography, magnetoencephalography, temporal fine structure (TFS), selective attention, new early filter model, cognitive hearing science

## Abstract

The rostral brainstem receives both “bottom-up” input from the ascending auditory system and “top-down” descending corticofugal connections. Speech information passing through the inferior colliculus of elderly listeners reflects the periodicity envelope of a speech syllable. This information arguably also reflects a composite of temporal-fine-structure (TFS) information from the higher frequency vowel harmonics of that repeated syllable. The amplitude of those higher frequency harmonics, bearing even higher frequency TFS information, correlates positively with the word recognition ability of elderly listeners under reverberatory conditions. Also relevant is that working memory capacity (WMC), which is subject to age-related decline, constrains the processing of sounds at the level of the brainstem. Turning to the effects of a visually presented sensory or memory load on auditory processes, there is a load-dependent reduction of that processing, as manifest in the auditory brainstem responses (ABR) evoked by to-be-ignored clicks. Wave V decreases in amplitude with increases in the visually presented memory load. A visually presented sensory load also produces a load-dependent reduction of a slightly different sort: The sensory load of visually presented information limits the disruptive effects of background sound upon working memory performance. A new early filter model is thus advanced whereby systems within the frontal lobe (affected by sensory or memory load) cholinergically influence top-down corticofugal connections. Those corticofugal connections constrain the processing of complex sounds such as speech at the level of the brainstem. Selective attention thereby limits the distracting effects of background sound entering the higher auditory system via the inferior colliculus. Processing TFS in the brainstem relates to perception of speech under adverse conditions. Attentional selectivity is crucial when the signal heard is degraded or masked: e.g., speech in noise, speech in reverberatory environments. The assumptions of a new early filter model are consistent with these findings: A subcortical early filter, with a predictive selectivity based on acoustical (linguistic) context and foreknowledge, is under cholinergic top-down control. A prefrontal capacity limitation constrains this top-down control as is guided by the cholinergic processing of contextual information in working memory.

## Introduction

One of the most challenging tasks that most people perform upon a daily basis is perceiving and understanding speech in background sound such as noise. Be that noise interfering voices in a restaurant, music, or traffic in the street, the socio-psychological impact is profound for many elderly listeners, whether or not they suffer from peripheral hearing loss. The majority of audiological patients have difficulty understanding conversation in noise (Kochkin, 2000). Noise may obscure or degrade speech information, such that only a fraction of the speech signal is available to the listener's brain. Listening and communicating under adverse conditions (Mattys et al., 2012) is known to engage compensatory brain mechanisms, particularly in elderly listeners (Wong et al., 2009).

The purpose of this article is to provide a theoretical model explaining phenomena related to the cognitive hearing science of the perception and comprehension of speech in noise. This model is intended to focus new enquiry. Having highlighted the scale of the problem motivating this objective, we first offer two necessary definitions: (i) Elevated audiometric thresholds define hearing impairment; (ii) Sensory processing is the way that the nervous system receives information from the auditory periphery and turns that information into perceptual representations. Deficits of sensory processing thus not only include losses that cause elevated audiometric thresholds and/or supra-threshold auditory processing deficits, but also include what has been termed “hidden loss” (Schaette and McAlpine, 2011; Plack et al., 2014). Considering such hidden loss, Kujawa and Liberman (2015) have revealed cochlear synaptopathy in an animal model, characterized by changes either at the level of the synapse from hair cells to auditory nerve fibers or at the level of the nerve fibers themselves. Kujawa and Liberman showed that in age-related hearing loss, synaptopathy precedes hair cell loss. This synaptopathy likely causes problems hearing in noise even before the loss of those hair cells. Accordingly, such synaptopathy is one origin of a hidden loss, which affects hearing (in noise) without elevating audiometric thresholds. Further, when the person's brain adapts to peripheral loss such as damage to hair cells, this loss can become hidden. The nervous mechanisms of sensory processing between primary auditory nerve fibers and the rostral brainstem of the central auditory system thus undergo adaptive neuroplastic changes, such that the individual is audiometrically normal (Schaette and McAlpine, 2011). The evidence for hidden loss thus challenges a watertight definition of hearing impairment based on audiometric thresholds alone. To further specify the definition of sensory processing, deficits in sensory processing may thus reside in the auditory periphery or in the central auditory system. However, the long-term neuroplastic changes in sensory processing, which accommodate sensorineural loss, involve adaptive changes in the auditory nerve and/or the central auditory system.

Turning from defining sensory processing to applying this notion to aging, the aging of individuals with bilateral sloping hearing loss causes a decline in sensory processing. Specifically, the weaker activation of superior temporal regions reflects that decline (Wong et al., 2010). This is accompanied by an increase in the recruitment of more general cognitive brain areas of the frontal lobe (Wong et al., 2009). The development of a larger and more active left pars triangularis of the inferior frontal gyrus and the left superior frontal gyrus compensate when listening under adverse conditions including speech in noise (Wong et al., 2010). Also, prefrontal activation correlated positively with improved speech-in-noise performance in older adults. These data thus support the decline-compensation hypothesis (Wong et al., 2009). This hypothesis postulates that the neurophysiological characteristics of an aging brain with respect to sensorily and cognitively demanding tasks include a reduced activation in (auditory) sensory areas, which otherwise support sensory processing, alongside an increase in general cognitive (association) areas, respectively. Long-term neuroanatomical changes, which permit compensatory prefrontal cortical activation to sensory decline, may be a double-edged sword. Such changes may cause maladaptive changes in cognitive abilities not related to speech-in-noise perception. In that sense, these changes would reflect a cognitive decline. Having introduced the decline-compensation hypothesis, we now turn to other extant hypotheses.

A seminal review (Schneider and Pichora-Fuller, 2000) contrasts four further hypotheses of associated declines in sensory and cognitive processing. The “sensory deprivation hypothesis” and the “information degradation hypothesis” both assume that sensory decline occurs before cognitive decline. The “sensory deprivation hypothesis” assumes that prolonged sensory decline drives a chronic cognitive change. By contrast, the “information degradation hypothesis” assumes that sensory decline immediately drives an acute cognitive decline. The “cognitive load on perception hypothesis” assumes that age-related cognitive decline occurs before sensory decline. Cognitive decline thus drives changes in perception: what we term sensory processing. The “common-cause hypothesis” assumes a common age-related factor causes a deterioration of both sensory processing and cognition. Wong et al.'s (2009, 2010) data supporting the decline-compensation hypothesis are also compatible with long-term chronic changes assumed by the sensory deprivation hypothesis. These data are not compatible with the acute changes assumed by the information degradation hypothesis and are agnostic as to whether sensory decline drives cognitive decline, or vice-versa as the cognitive load on perception hypothesis assumes. However, these data out-rule the common-cause hypothesis: There was not an age-related decline in the activation during speech-in-noise perception across sensory and cognitive areas (Wong et al., 2009).

Pertinent to these findings, Lin et al. (2011) postulated that the compensatory dedication of general cognitive resources to difficult auditory perception could also cause an accelerated decline in cognitive faculties. With peripheral age-related hearing loss leading to deafferentation of the auditory nerves and, in turn, a loss of afferents within the central auditory system, what happens is that the perception and understanding of speech becomes more difficult. Other cases where auditory perception is difficult are under environmentally adverse conditions such as noise or reverberation. A competing theory that Lin et al. evaluated is that social isolation and loneliness, caused by communication impairments (Strawbridge et al., 2000), could relate to cognitive decline and neuroanatomical indicators of Alzheimer's disease pathology (Bennett et al., 2006). The decline-compensation hypothesis (Wong et al., 2009, 2010) rather assumes that the compensatory dedication of general cognitive resources to difficult auditory perception accelerates neurocognitive decline. Of particular interest are complex span tests that assess working memory capacity (WMC); (e.g., Daneman and Carpenter, 1980; Turner and Engle, 1989; for an introduction to different working memory (WM) processes, see Baddeley, 1986). These complex span tasks involve retaining a memory load during some form of concurrent mental processing—tasks that are more strongly affected by cognitive aging than simple verbal short-term memory span (Bopp and Verhaeghen, 2005). Forward digit span requires the mental operations of retaining digit items in their original order, a measure of simple verbal short-term memory span. Backward digit span also requires the concurrent reordering of those items for backward report. Backward digit span and complex span tasks thus share the common requirement for concurrent mental processing during retention. Backward recall, sharing commonalities with both forward recall and complex span, is thus only intermediately susceptible to cognitive aging (Bopp and Verhaeghen, 2005).

Having introduced aging and working memory, it is worth considering the role of working memory in the perception of speech under acoustically adverse conditions. Perceiving and understanding speech in noise involves retaining a memory load. Such context proactively predicts, and retroactively repairs, utterances containing degraded sensory information (Marslen-Wilson, 1975; Samuel, 1981; Shahin and Miller, 2009; Shahin et al., 2012). The retention of information occurs while the listener concurrently performs linguistic processing. This lingustic processing affects the perceptual and semantic processing of that degraded sensory information in a top-down manner. Indeed, Uslar et al. (2013) revealed that the more complex the linguistic processing required, when perceiving speech in noise, the higher the signal-to-noise ratio required to identify 80% of the presented stimuli. Uslar et al.'s findings thus cohere well with the notion that speech-in-noise perception relies on a WM function: managing the trade-off between the (more complex linguistic) processing and the retention of (semanto-syntactic contextual) information. Further, corroboration of this notion stems from training on a backward span task (in noise). Such training improves complex span performance—WM improvements generalizing from the backward span task—and also enhances speech-in-noise performance (Ingvalson et al., 2015).

Turning to a different form of adverse conditions, background noise from to-be-ignored sources is not the only form of noise affecting the processing of to-be-attended speech. Reverberation pervades the built-environment and is particularly challenging for hearing-impaired listeners: The speech signal produced by the talker reverberates-off of hard surfaces, such as walls, reaching the listener in the form of an echo at a delay from the speech signal. Reverberation thus obscures speech perception cues of the direct signal (Nábělek, 1988). However, it has been shown that humans have the ability of perceptual compensation (Watkins and Raimond, 2013): They use tacit knowledge of the room acoustics from immediate prior speech sound context to reduce the adverse effects of reverberation on speech perception. Accordingly, the listener's brain forms, and retains in memory, a mental model of the room's acoustics when listening. This model is used in a top-down manner to select and predict the perceptual representation of the current utterance to support speech perception under reverberatory adverse conditions.

A goal of the present article is thus to refocus new enquiry into the perception and comprehension of speech under adverse conditions by offering a new theoretical cognitive model of subcortical speech processing. The necessary evidence integrated thus centers on the relation of WM to the brainstem's processing of speech under adverse conditions. These conditions include noise and reverberation. A further goal is to communicate, beyond the consequences of such peripheral masking effects, how cognitive aging and plasticity of the auditory nerves and central auditory system driven by hearing loss can affect the brain's processing of speech in noise.

In the following, we will introduce the pivotal role of the rostral auditory brainstem as an anatomical and informational hub of the “bottom-up” ascending and “top-down” descending auditory systems. In turn, we will review the current state-of-the-art on the complex Auditory Brainstem Response (cABR) to speech sounds. What then ensues is a discussion of findings concerning the relation of effects of reverberation on the speech intelligibility to the speech ABR representation of speech TFS. These findings concern elderly listeners. This discussion will flow then into how memory load and WMC can influence the generation of wave V of the auditory brainstem response (ABR) to clicks. In turn, the influence of memory load and sensory load on auditory distraction will be considered. The discussion will ultimately converge on a new early filter model, reviving Broadbent's (1958) influential assumption: There is a capacity limitation on how the human mind processes information. That bottleneck in processing selects information early on for further processing. The rostral brainstem is arguably crucial in the operation of that early filter, to which we now turn.

## The rostral brainstem as a computational hub in the ascending and descending auditory systems serving as an early filter

### Generators of the auditory brainstem response

**Figure 1:**
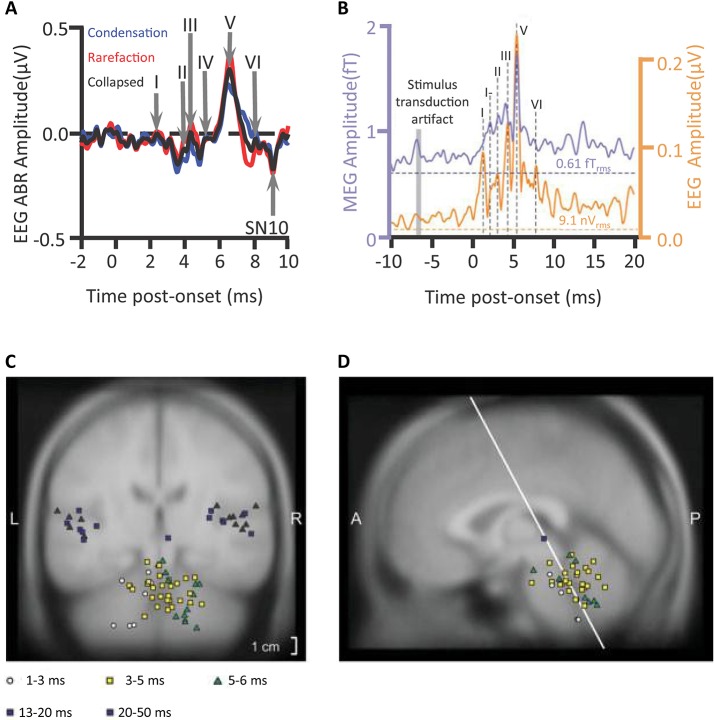
**Auditory brainstem response deflections**. An individual's auditory brainstem responses (ABRs) averaged from waveforms of scalp-measured electroencephalogram (EEG) epochs in response to clicks **(A)** plotted for condensation or rarefaction leading phase or collapsed across leading phase. Waves I–VI are visible as positive deflections at the scalp. A subsequent scalp negativity, which though reduced by a filter, is still visible (SN10), Campbell et al. (2012); *n* = 1. The grand-averaged wave V latency-normalized ABR to clicks presented to the left ear and the average of corresponding magnetic ABR waveforms (mABRs) was acquired simultaneously with a whole head array of magnetometers and collapsed across magnetometers **(B)**, Parkkonen et al. (2009); *n* = 7. Equivalent Current Dipoles **(C,D)** locations were normalized from individual MRIs onto the coordinates of the Montreal Neurological Institute average brain offering theoretical source generators of mABR deflections, wave V (green triangles) being generated contralateral to stimulation. SN10 generators and auditory middle latency generators localized to cortical regions. *Credits:* **(A)** is adapted with permission from Campbell et al. (2012). Promotional and commercial use of the material in print, digital or mobile device format is prohibited without the permission from the publisher Wolters Kluwer Health. Please contact healthpermissions@wolterskluwer.com for further information. **(B–D)** are adapted with permission of John Wiley and Sons from Parkkonen et al. (2009). Copyright © 2009 Wiley-Liss, Inc.

**Figure 2:**
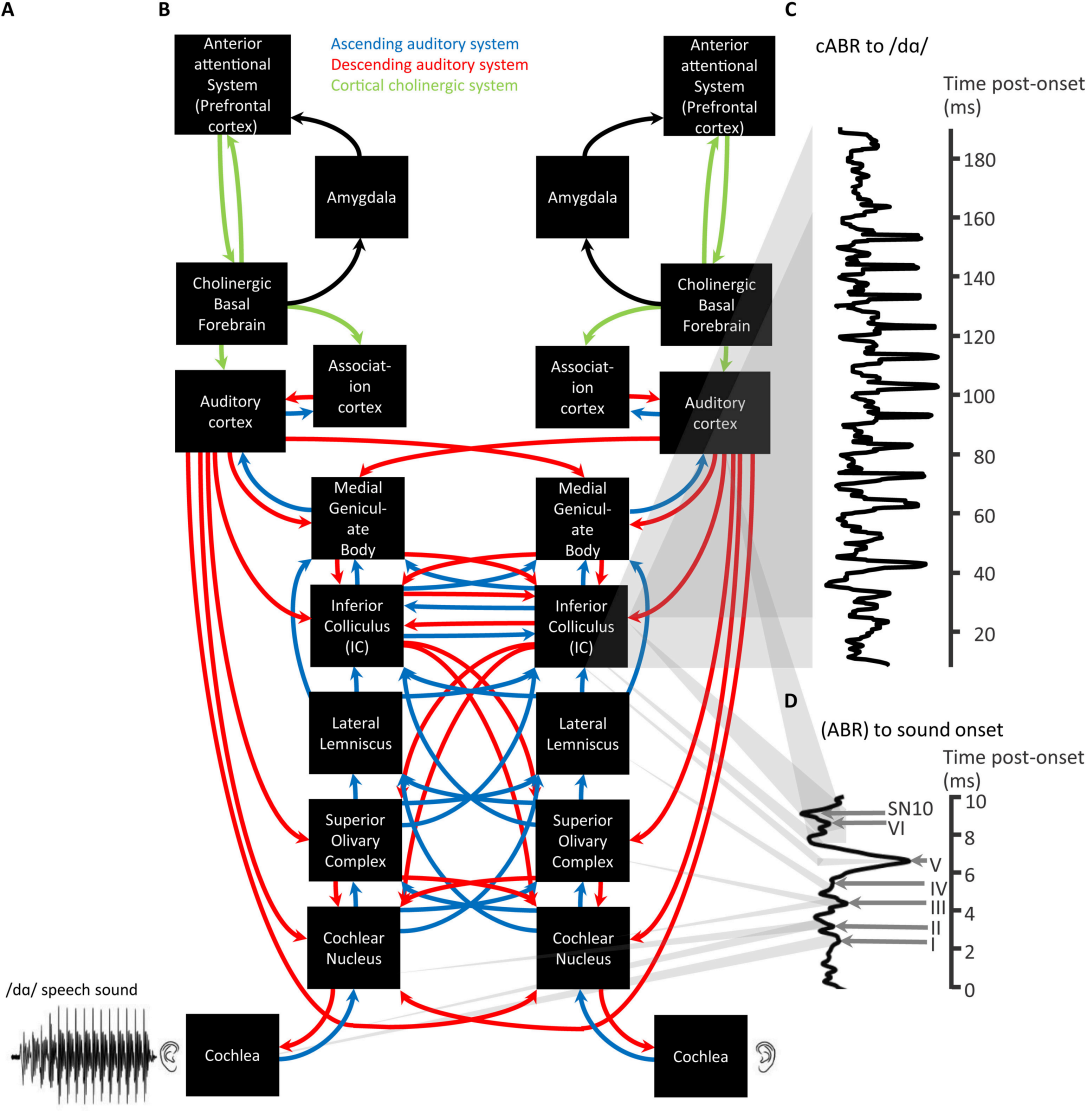
**A schematic of cortical cholinergic influence, reliant on the neurotransmitter acetylcholine, on the descending auditory system affecting the flow of information through the ascending auditory system in relation to the generation of ABRs and cABRs**. A complex stimulus waveform of a speech sound /dɑ/, illustrated on the lower left **(A)**, passing through the rostral brainstem including the Lateral Lemniscus and Inferior Colliculus **(B)** generating a cABR **(C)**. As also shown in green **(A)**, these structures are under the top-down control of the prefrontal cortex via basal forebrain cholinergic projections (green) to auditory cortex that corticofugally control corticopetal-corticofugal loops in the ascending (blue) and descending (red) auditory system. These loops thus attentionally tune the selective processing of ascending auditory information. There is a delay in the time-course of the cABR with respect to the stimulus waveform by the time (ca. 8 ms) for ascending auditory information to reach the rostral brainstem. The preceding ABR to stimulus onset is generated during this delay **(D)**. Gray shadowing denotes theoretical mappings of source generators to scalp-measured responses; *n* = 21. *Credits:* The schematic of the ascending and descending auditory pathways is adapted with permission of John Wiley and Sons from Chandrasekaran and Kraus (2010). Copyright © 2010 Society for Psychophysiological Research. Waveforms are reprinted from Chandrasekaran et al. (2009), Copyright © 2009, with permission from Elsevier in respect to Chandrasekaran et al. (2009: Exp.1).

A rapid volley of deflections of the click-elicited ABR, deflections of scalp-measured electrical potentials, occur mostly within the first 10 ms after the onset of a sound (Figure 1A). Tone-pip-elicited ABR deflections occur slightly later (Ikeda, 2015). Assessments of the deflections of ABRs are already in routine clinical use. The audiology lecturer's E-COLI mnemonic (Hall, 2007) detailing a one-to-one peak-to-structure mapping, misidentifies the nature of ABR source generation. The mnemonic specifies E: eighth nerve action potential (wave I); C: cochlear nucleus (wave II); O: olivary complex (superior) (wave III); L: lateral lemniscus (wave IV); I: inferior colliculus (wave V). This bottom-up route does reflect some of the detail of the ascension of information through the subcortical auditory system upwards toward the medial geniculate body of the thalamus and then the auditory cortex. Yet, sophistication is warranted: Many-to-one mappings of anatomical source generator structures to each deflection are apparent (Hall, 2007). Further, vertex-negative troughs as well as vertex-positive peaks can also have source generators. Multiple sources can be concurrently active and a subset of those generators reflected in the timing and amplitude of the ABR peak (Figures 2A,B).

Further vindicating a sophistication concerning the mapping of source generators to deflections, a far-field magnetoencephalographic investigation (Parkkonen et al., 2009) localized wave V to regions posterior and lateral to both the lateral lemniscus and inferior colliculus (IC) of the hemisphere contralateral to the stimulation. These Equivalent Current Dipole source models of magnetic Auditory Brainstem Responses (mABR) represented the net effect of simultaneously active sources. It cannot be out-ruled that concurrent activation of both lateral lemniscus and IC contributed to this Wave V. However, as measured directly during surgery, fibers of the lateral lemniscus have been shown to generate the Wave V peak (Møller and Jannetta, 1982; Møller et al., 1994). Those fibers enter the IC, though there may be further consequences for the activation of the IC indicated by the later longer-lasting high-amplitude SN10 negativity (Davis and Hirsh, 1979; Møller and Jannetta, 1983)^1^. This IC is the largest structure of the brainstem and wave V the largest wave of the ABR with commonly used filtering parameters. However, wave V is not affected by deafferentation of the IC (Møller and Burgess, 1986).

As depicted in Figure 2A, ABR source generators are subcortical processing stations. These stations are on the pathway of the ascending auditory system, mediated by neuronal elements originating from sensory receptors. In psychological terms that pathway may be described as bottom-up. This pathway begins with the auditory nerve fibers that input the cochlear nuclei and bifurcate from where information is then transmitted upward to other brainstem, midbrain, and thalamic stations up to the auditory cortex. These ascending connections running from cochlear to cortex are termed corticopetal connections.

#### Interim summary

There are a series of subcortical generators of the ABR within the ascending auditory system. There are many-to-one mappings from the activation of generators to the sequence of scalp-measured deflections in the ABR.

### Corticopetal-corticofugal loops

Not only is there an ascending auditory system, as we have already introduced, but there is also a descending auditory system. There are extensive efferent top-down projections of this descending auditory system. These systems of ascending and descending connections are not independent (Bajo and King, 2013). Rather, Bajo and King theorize that the auditory system is a series of dynamic loops in which changes in activity at higher levels in the brain affect neural coding in the IC. These loops also affect other subcortical nuclei as much as signals received from lower structures of the brainstem (Figure 2). In control theory, such loops could permit a corrective positive feedback. Accordingly, a loop receives a top-down expectancy of neural output descending from the requirements of higher structures of the auditory system. To specify these terms, an “expectancy” is a prediction signal from higher structures to lower structures in the context of previous ascending input from a lower structure. This prediction signal is also based on what information the higher structures “require” lower structures to select. For instance, consider selective attention to behaviorally relevant targets of a certain fundamental frequency: The prediction signal coding the expectancy from higher structures may require lower structures to provide information about the behaviorally relevant fundamental frequency. The deviation of the actual neural output of an ascending connection from that expectancy then leads to an alteration in the descending connections of that loop. Those altered descending connections, in turn, affect how the ascending connections code future neural input. As Figure 2 depicts, the auditory system is thus theoretically a collection of dynamic control loops. As each of these loops contain corticopetal and corticofugal connections, such a loop is termed a corticopetal-corticofugal loop. Each loop is influenced by changes in higher levels and input from lower loops. Suga et al. (2000) postulate that such corticopetal-corticofugal loops perform cortically “egocentric selection.” Noise information ascends affecting descending corticofugal connections. This effect on corticofugal connections leads to a transient shift, thus sharpening the lateral inhibition of ascending connections. Accordingly, subsequent noise leads to a small suppressed ascending output to noise information: a small short-lived cortical change thus occurs in response to noise stimulation. When the ascending information is a fear-conditioned signal rather than noise, that information ascends to the auditory cortex and auditory association cortex. In turn, these cortices activate the cholinergic basal forebrain via the amygdala—a cortical influence on the basal forebrain that can also be affected by an unconditioned somatosensory shock stimulus, possibly by ascending thalamic routes (Weinberger, 1998).

#### Interim summary

The auditory system is a hierarchy of corticopetal-corticofugal loops. These loops can dynamically adapt. By virtue of being hierarchically organized, such a loop can selectively filter incoming information on the basis of top-down control from higher structures.

### Cortical cholinergic attention system

**Figure 3:**
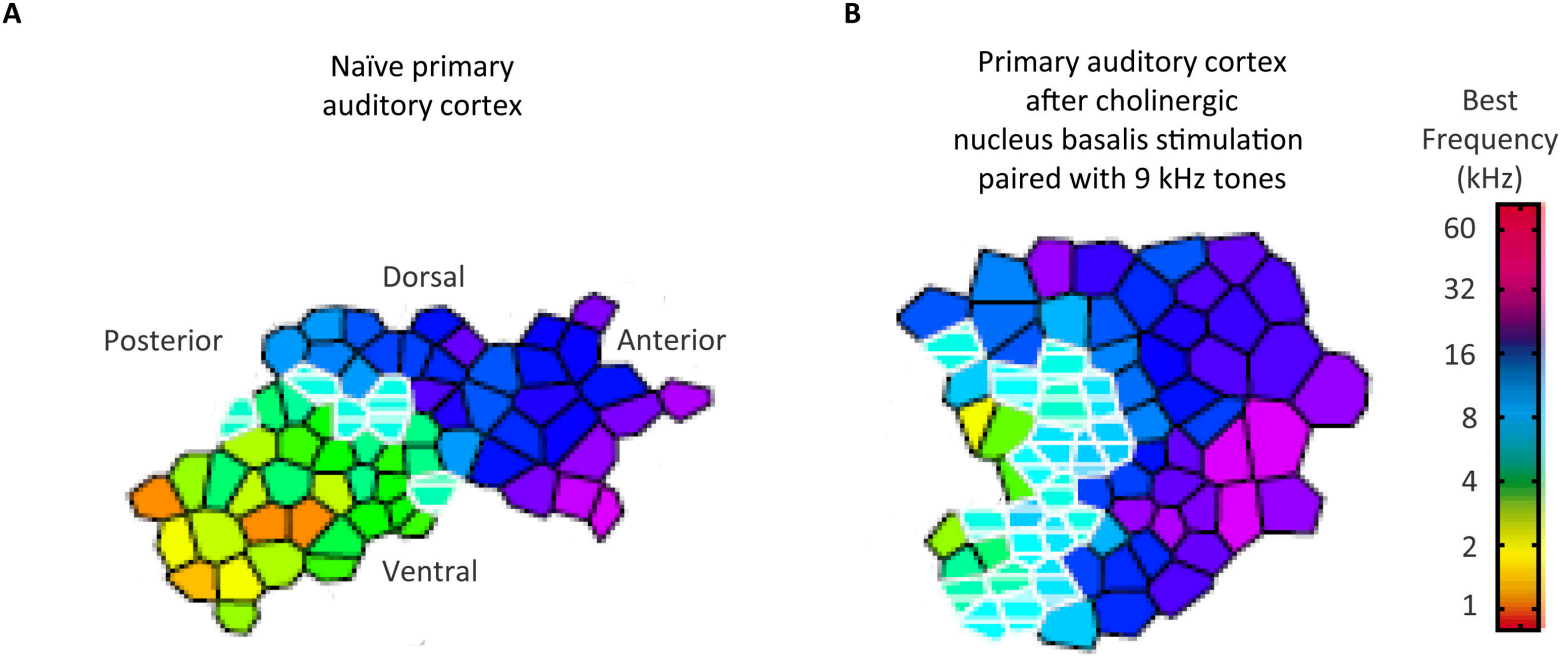
**Cholinergic influences on the auditory cortical organization without fear conditioning**. The representative best frequency map, derived from cortical mapping of respones to pure tones of 45 different frequencies at 15 different intensities, show tonotopy of naïve primary auditory cortex **(A)** and the corresponding map following the pairing of 9 kHz sounds with stimulation of the cholinergic nucleus basalis of the basal forebrain **(B)**. A comparable auditory cortical reorganization did not occur with pairing of sounds with such stimulation after a lesioning of cholinergic rather than GABAergic nucleus basalis neurons. *Credit:* From Kilgard and Merzenich (1998). Reprinted with permission from AAAS.

Having introduced the notion of hierachical control of corticopetal-corticofugal loops of the central auditory system, we turn now to how the highest of these loops could be controlled. Sarter et al. (2005) reviewed evidence for a reciprocal feedback loop between the basal forebrain and the prefrontal cortex. This feedback loop controls the cholinergic projections to the prefrontal cortex within an anterior attentional system (Figure 2A). This positive feedback loop also controls the cholinergic output to other brain areas including sensory areas, yet without reciprocal feedback. Such a system of cholinergic feedback has the basis for top-down control of sensory processing. This control occurs through the basal forebrain through the release of acetylcholine by efferent top-down projections to sensory areas including the auditory cortex (Kilgard and Merzenich, 1998; Figure 3). Acetylcholine thus affects the auditory cortex; top-down projections influencing sensory cortical processing. Kilgard and Merzenich revealed that such top-down reorganization occurred without either a fearful or an aversive stimulus. It is thus viable that prefrontally controlled attention to stimuli, for instance during the long-term experience of listening to a specific language, rather than fear conditioning, can cholinergically permit attention to those auditory experiences to cause long-term changes in the operation of egocentric selection by corticopetal-corticofugal loops. Also viable is that the prefrontally controlled cholinergic modulation of corticofugal connections from the auditory cortex is an attentional modulation of auditory subcortical processing.

Visual attentional demands can also influence such subcortical auditory processing. When a cat visually attends a mouse, subcortical auditory responses of the dorsal cochlear nucleus are reduced (Hernández-Peón et al., 1956). Further, attention to a visual discrimination task reduces responses of the auditory nerve to clicks (Oatman, 1971; Oatman and Anderson, 1977). In humans, Lukas (1980) revealed that attention to the visual modality also reduces auditory nerve responses, while Puel et al. (1988) showed that such attention reduced the otoacoustic emissions evoked by a click. Prefrontal influences of visual attention on such subcortical auditory filtering by corticofugal influences on corticopetal-corticofugal loops could also, in turn, permit visual attention to influence the cortically generated auditory supratemporal mismatch negativity (Erlbeck et al., 2014; Campbell, 2015). This convergent evidence thus points toward a very early stage of attention that influences subcortical auditory mechanisms.

#### Interim summary

We introduced the cholinergic top-down control assumption that the cholinergic cortical attentional system controls an early filter. Corticofugal modulation of corticopetal-corticofugal loops leads to an attentional selection crucially affecting the level of the rostral brainstem. The rostral brainstem is the locus of action of that filter, being integral to the confluence of ascending, descending, ipsilateral, and contralateral effective connectivity of the subcortical central auditory system.

### Attention and auditory brainstem responses

In contrast to this evidence for top-down control, ABRs proved, in several early studies, to be unaffected by attention (Woldorff et al., 1987, 1993; Woldorff and Hillyard, 1991). Compelling was that, juxtaposed with Woldorff et al.'s findings indicating there are no attentional effects on ABRs, in the same studies, there were attentional augments of auditory middle latency response (AMLR) deflections (20–50 ms.), alongside attentional augments of auditory long latency responses (ALLRs). These ALLRs include N1 and P2. In Woldorff et al.'s “dichotic” listening tasks, participants attempted to attend to target deviants (*D*) in an oddball sequence of standards (*S*), *SSSSDSSSSSSSSD…* Attending those deviants, while ignoring unattended deviants in an oddball sequence, presented in the other ear, affected the P20–P50 of the AMLR and the “Nd” of ALLRs. Contrastingly, ABRs were unaffected by such attention in these dichotic listening tasks.

**Figure 4:**
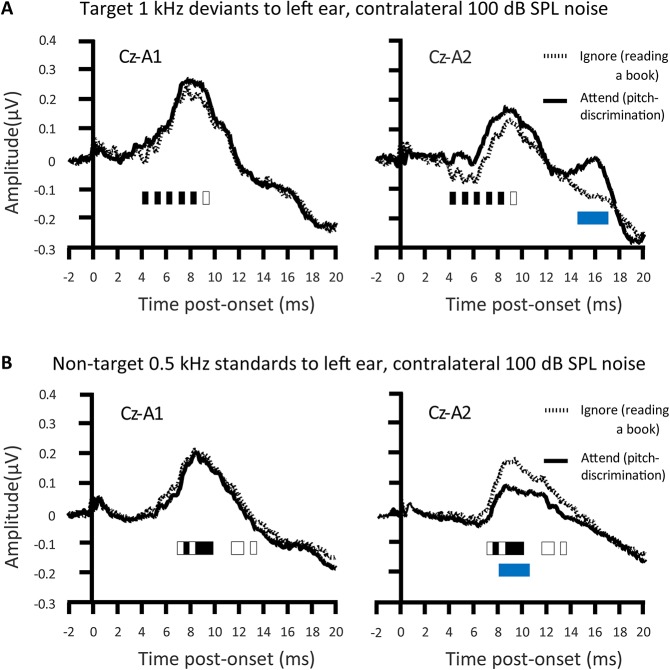
**Attention modulations of the auditory brainstem response (ABR)**. An attentional augment overlaps the grand-averaged ABRs to deviants presented with contralateral 100 dB SPL noise. That augment is a vertex positivity occuring when participants attend for deviant targets, relative to when participants ignored the sounds whilst reading a book **(A)**. This augment reached significance at the times denoted by black rectangles. The response to the attended target was significantly more contralateral during the time denoted by the blue rectangle. There was a corresponding attentional decrement, a negativity, to the attended non-target standards, relative to when participants ignore all sound whilst reading a book **(B)**. This decrement reached significance at the times denoted by black rectangles. This attentional decrement was also significantly more contralateral during Wave V, as denoted by the blue rectangle; *n* = 24. *Credit:* Adapted with permission from Ikeda et al. (2008). Promotional and commercial use of the material in print, digital or mobile device format is prohibited without the permission from the publisher Wolters Kluwer Health. Please contact healthpermissions@wolterskluwer.com for further information.

Inconsistent with the findings of Woldorff et al., Ikeda et al. (2008) showed that selective attention affected tone-pip ABRs (Figure 4). A task requirement of perceptual discrimination between pips of a target frequency and a non-target frequency, alongside rather loud (100 dB SPL) contralateral masking noise, sufficed to cause attentional augments of ABRs. Those attentional augments occurred in the range of waves II–VI in response to attended target sounds relative to sounds that participants just ignored (while reading a book). Conversely, Ikeda et al. (2008) also revealed attentional decrements of all ABRs to attended frequent non-targets relative to acoustically identical sounds that participants just ignored. The augments and decrements of ABRs by selective attention were particularly apparent with a contralateral Cz-A2 bipolar channel than with the Cz-A1 channel ipsilateral to stimulation. These Cz-A2 ABRs arguably more strongly reflected right hemisphere generators that were contralateral to the left ear that received the tone pips. The extent of these selective attention effects on ABRs were also stronger with louder (100 dB SPL) than with quieter (80 dB SPL) masking noise. The implication is that the mechanisms of selective attention affecting ABR generation are promoted by the binaural interaction of information from to-be-ignored masking noise; masking noise that would make the task more effortful. These mechanisms affect generators ipsilateral and contralateral to the attended ear. An assumption is that these mechanisms involve the descending corticofugal routes between subcortical processing stations.

The earliest signs of binaural interaction of the ascending auditory system in the ABR, at least in some individuals, occur during Wave III (e.g., Wong, 2002; Hu et al., 2014). This Wave III generation could implicate the superior olivary complexes (SOC) after the first bifurcation from the cochlear nucleus within the subcortical ascending auditory system. Such binaural interactions can be attentionally modulated at least for tone-pip stimuli (Ikeda, 2015). These interactions involve cells exhibiting ipsilateral excitation alongside contralateral inhibition (Ikeda, 2015). Conceivable is that binaural interactions with tone pips also engage cells exhibiting ipsilateral excitation as well as contralateral excitation (Ikeda, 2015). The findings of Ikeda et al. (2008) revealed that selective attentional effects on Wave II can be affected by contralateral noise. The descending olivocochlear projection could mediate an improved selection of the attended target at the level of the cochlear nucleus. This selection would occur prior to the first bifurcation of the ascending auditory system including an ascending projection to the SOC of the contralateral hemisphere. The top-down influence of that descending olivocochlear projection could exclusively involve covert attentional mechanisms. Such mechanisms could operate at the level of the cochlear nucleus or also involve the outer hair cells (Maison et al., 2001). Another hypothesis is that these covert attentional mechanisms even modulate the muscles affected during the middle ear acoustical reflex (Ikeda et al., 2013). There is thus evidence for a corticofugally operated top-down early selective filtering mechanism affecting processing during the first few milliseconds. This mechanism comes particularly into play under adverse conditions including noise (Maison et al., 2001). This mechanism is arguably less necessary and apparent under the experimental conditions that Woldorff et al. employed. The early processing of that sound, affected by top-down attentional effects, thus becomes sensitive to the demands of the task and what the sound is.

#### Interim summary

The ABR is attentionally modulated in loud noise.

### Refractoriness of ABRs and ALLRs

**Figure 5:**
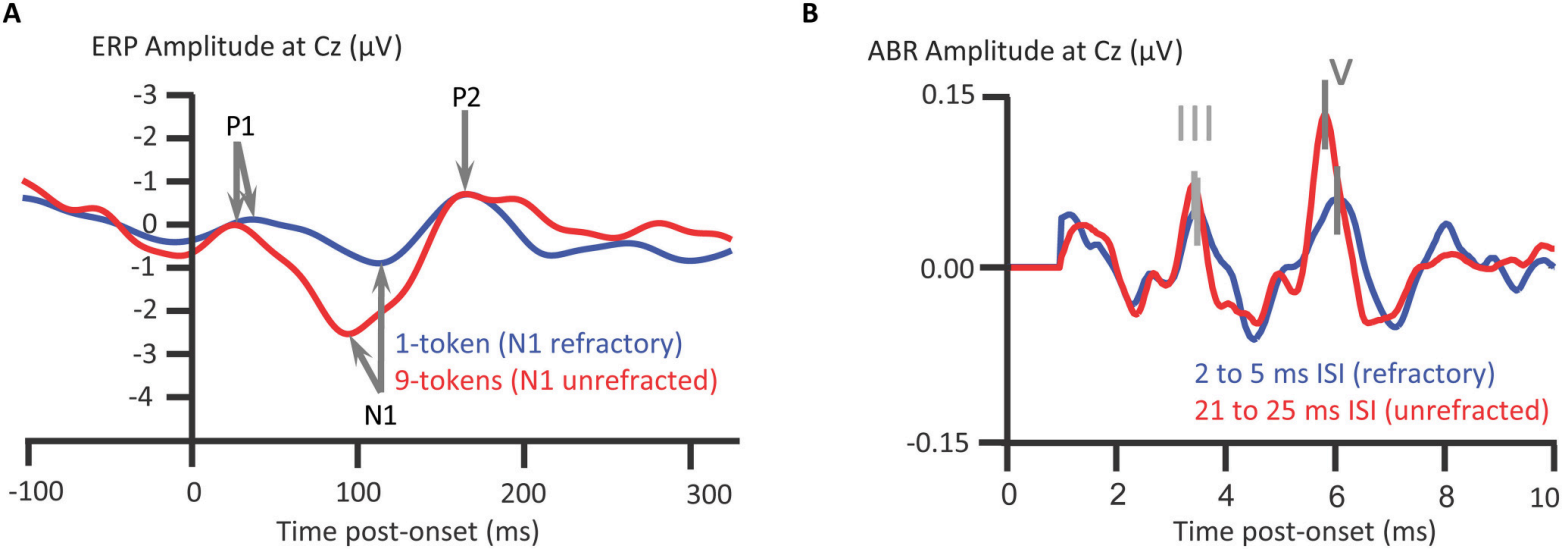
**Longer refractory periods of auditory N1 than for ABRs**. The grand-averaged auditory N1 to a tone in a pitch-varying sequence of tones, 9 different pitch tokens, presented at an interstimulus interval (ISI) of 328 ms., is less refracted than when presented in a 1-token repeated tone sequence **(A)**. Stimulus-specific cortical neuronal elements sensitive to pitch become less responsive upon repeated stimulation, as recovers after a period of quiescence. Such quiescence is more common with multiple different pitch tokens. The inter-token repetition interval between stimulation of stimulus-specific elements is longer with a higher token set size. Such elements contribute to N1 generation and thus N1 is refracted in the 1-token relative to 9-token sequences; Campbell et al. (2007); *n* = 12. ABRs are also subject to refractoriness **(B)**, though new deconvolution techniques show sounds still elicit ABRs with ISIs of 2–5 ms. The ABRs are from a representative participant with intact hearing, Valderrama et al. (2014); *n* = 1. *Credits:* **(A)** is adapted with permission of John Wiley and Sons from Campbell et al. (2007). Copyright © 2007 Society for Psychophysiological Research. **(B)** is reprinted from Valderrama et al. (2014). Copyright © 2014, with permission from Elsevier.

We turn now from attentional modulations of ABRs and ALLRs to their relative susceptibility to attenuation on repeated presentation of a sound: refractoriness. In this subsection, we intend to tackle why the subcortical processing indexed by ABRs more closely reflects temporal information within the acoustical waveform than thalmocortically generated responses. The answer to this question hinges on this notion of refractoriness. The time-course of auditory evoked responses (EPs), otherwise known as “auditory event-related potentials” (ERPs), are time-locked to the onset of a sound. Deflections of the ALLRs of auditory ERPs, such as the supratemporally generated auditory N1, attenuate on repeated presentation of a sound. This attenuation recovers after a period of silence (e.g., Butler, 1973; Campbell and Neuvonen, 2007), as is termed the refractory period. When stimulus-specific neuronal elements are unstimulated, those neurons are released from refractoriness (e.g., Campbell et al., 2003, 2005, 2007; see Figure 5A). By contrast to ALLRs, such as the auditory N1, ABRs are relatively unaffected by refractoriness: For instance, even with multiple reductions in interstimulus interval from 53 to 3 ms, all ABR deflections were unaffected except for wave V (Picton et al., 1992). Wave V showed a prolongation of peak latency at interstimulus intervals of 3 ms only. However, Valderrama et al. (2014) compared ABRs elicited with interstimulus intervals of 21–25 ms to those elicited with interstimulus intervals of 2–5 ms. Valderrama et al. thus found shorter interstimulus intevals reduced ABR amplitudes and affected ABR morphology. On balance, ABRs are less subject to refractoriness than the auditory N1; this refractoriness occuring at briefer interstimulus intervals, with which stimuli evoke ABRs with a clear morphology. Indeed, Valderrama et al. (2014) deconvolved overlapping ABR signals with interstimulus intervals as short as 2–5 ms. Thus when a complex sound such as a speech stimulus /dɑ/ is presented, the consequence, after the ABR to the onset, is that the rostral brainstem generates an ongoing response to aspects of the ongoing /dɑ/ sound.

#### Interim summary

The ABR is relatively unaffected by refractoriness. Thus when complex sounds are presented, a flowing river-of-information passes through the rostral brainstem that abstracts envelope and periodicity information generating a cABR response.

### Attention, expectancy, and prediction affect both the cABR and speech-in-noise perception

Being relatively unaffected by refractoriness, the cABR is thus responsive to landmarks in the acoustical waveform (Skoe and Kraus, 2010; Campbell et al., 2012). The representation of lower frequencies of that acoustical waveform predominates the cABR waveform. The cABR generator process thus seems to abstract the envelope and the fundamental frequency of the stimulus away from the acoustical waveform. The cABR does so at a time-lag of 8 to 10 ms. After the ABR response to the consonantal onset of the /dɑ/ stimulus, the cABR reflects that informational flow through rostral brainstem generators of the ABRs, with the contribution of a distinct Frequency Following Response or “FFR” (Chandrasekaran and Kraus, 2010; Xu and Gong, 2014; Bidelman, 2015; Xu and Ye, 2015). This FFR locks primarily to the fundamental frequency of the vowel portion that the rostral brainstem also generates, albeit in the IC. The form of FFR typically recorded when analyzing cABRs is an “envelope FFR” (Aiken and Picton, 2008) or “envelope following response” (Easwar et al., 2015; Varghese et al., 2015). This EFR follows the periodicity envelope. The envelope differs from the spectral FFR (Aiken and Picton, 2008; Easwar et al., 2015) that follows the spectral frequency of the stimulus. Though there are cochlear nucleus (CN), trapezoid body, and superior olivary complex (SOC) contributions to the FFR (Marsh et al., 1974) as well as a cortical contribution (Coffey et al., 2016), there is a dramatic reduction in a form of FFR accomplished by a subcortical cooling of the IC (Smith et al., 1975). On balance, generators in the vicinity of the rostral brainstem, encompassing the lateral lemniscus and IC, predominate both the cABR to consonantal and vowel portions of a speech sound. The flow of information through the rostral brainstem indexed by the cABR is time-lagged. This time-lag concerns how long the landmark information takes to reach the rostral brainstem. A series of investigations revealed that attention augments the FFR: Galbraith and colleagues (Galbraith and Arroyo, 1993; Galbraith et al., 1995, 1998, 2003) showed that whether comparing attending sounds to not attending sounds, or whether attending to a selected auditory stream of sound while ignoring another, an attentional augment of the FFR is shown and that FFR is higher in amplitude with speech sounds (for an alternative perspective, see Varghese et al., 2015). A separate series of experiments also corroborated that the familiarity of speech or music affected the time-course and dynamics of FFR via experience-dependent plasticity (Musacchia et al., 2007; Wong et al., 2007; Song et al., 2008; Chandrasekaran et al., 2012).

Turning from these initial studies revealing influences of experience and attention on FFRs, a recent investigation of auditory attention and FFRs (Lehmann and Schönwiesner, 2014) showed that attentional selection in background speech noise can rely on both frequency and spatial cues. This selection can also rely on frequency cues alone. In Lehmann and Schönwiesner's procedure, participants attended to vowels uttered by the designated speaker while ignoring another speaker (attend the male and ignore the female, or attend the female and ignore the male). These participants were required to detect occasional attended pitch-deviant target vowels by pressing a button. In a diotic condition, audio-recordings of a male repeating /a/ and a female speaker repeating /i/ were intermixed such that the same sound mixture was presented to both ears. In a dichotic condition, the male speaker's repeated /a/ was presented to the left ear and the female speaker's repeated /i/ was presented to the contralateral ear. In both the diotic and dichotic conditions, the FFR followed the distinct fundamentals of both vowels. In the dichotic condition only, attending the male (on the left) relative to attending the female (on the right) increased the amplitude of the FFR at the fundamental frequency of male's /a/. The direction of attention thus arguably affects the FFR. Lehmann and Schönwiesner computed a neural spectral modulation index of how much attention affects the FFR. This index was higher in the dichotic than the diotic conditions. Spatial cues were thus important to attentional selection, which conceivably occurs at the level the rostral brainstem. Further, frequency cues were also sufficient for attentional selection in that the modulation index was above zero in the diotic condition. Accordingly, attentional selection does not require the segregation of attended and ignored information to different sides of the brain. Further, individual variability in the amplitude of these attentional FFR augments, whilst selecting one voice and ignoring another, was related to the detection of pitch-deviant targets in the attended stream (Lehmann and Schönwiesner, 2014): the stronger the attentional modulation of FFR, the lower the discriminability of the attended pitch-deviant target. Relative to individuals performing at ceiling, participants who struggled more with the task thus applied more attention to the task's stimuli affecting the brainstem representation of those stimuli. The IC, at least in part, generated this attentionally augmented FFR (Bidelman, 2015). In addition, an extensive corticofugal efferent system arguably influenced the generation of this attentional augment in a manner that is both goal-directed and behaviorally relevant. For evidence of a cortical contribution to FFR, see Coffey et al. (2016).

**Figure 6:**
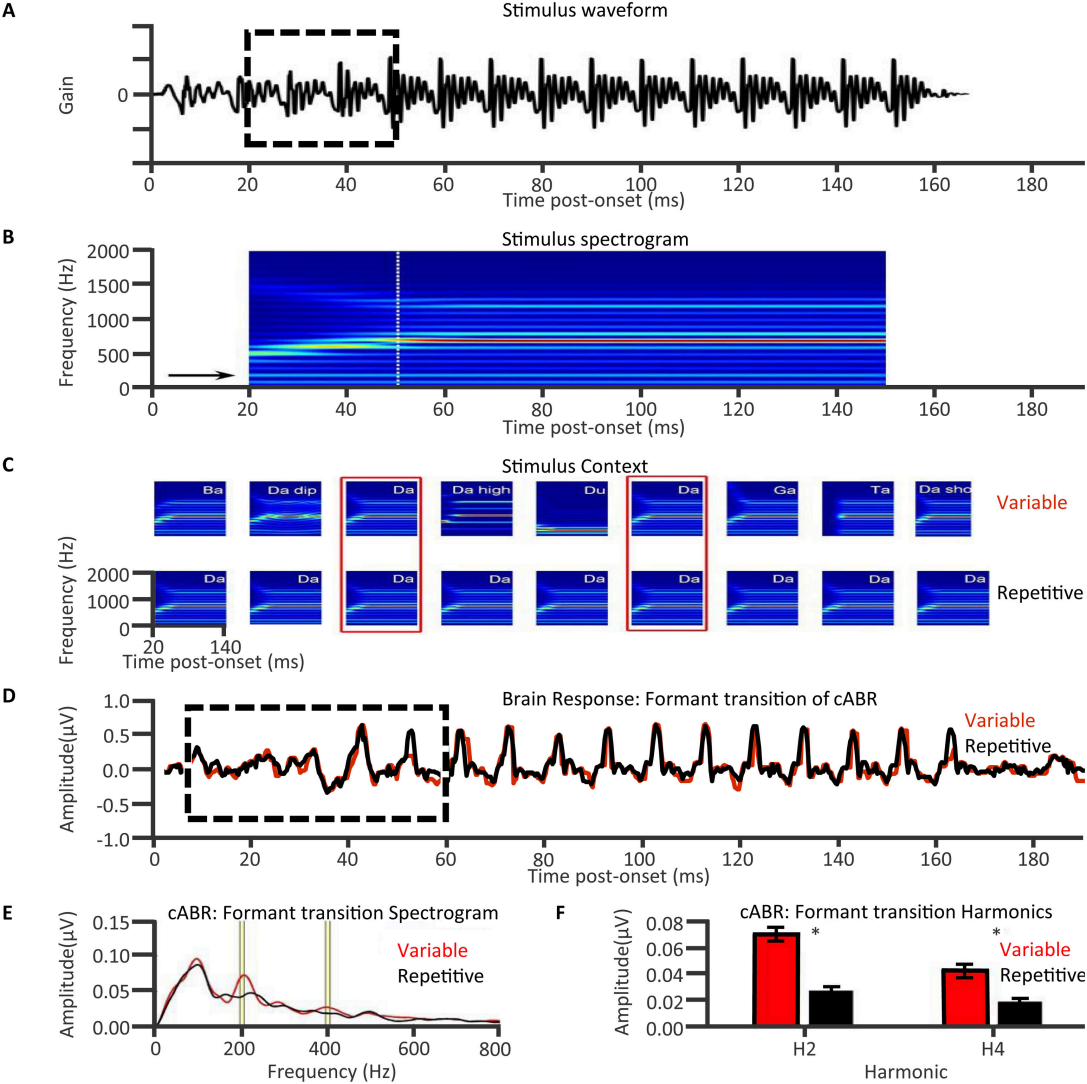
**Top-down influences of auditory speech context on cABRs: Speech stimulus context affects the formant transition of the cABR in a manner that predicts speech in noise performance in 8- to 13-year olds with intact hearing**. A long /dɑ/ stimulus **(A)** contains a formant transition (boxed) as the acoustical spectrogram **(B)** illustrates. Chandrasekaran et al. (2009) derived cABRs to /dɑ/ in a variable speech or in a repeated /dɑ/ context **(C)**. The cABRs revealed no significant effect of speech context during the steady-state vowel portion **(D)**, but during the formant transition (boxed) context influenced cABRs. The amplitude spectra of the cABR **(E)** during the formant transition revealed a repetitive context augmented the second and fourth harmonics, as was significant **(F)**. Correlations revealed that the higher this presumably top-down speech-context modulation of the representation of the second harmonic during the formant transition of the cABR, the better the speech-in-noise performance on the Hearing in Noise Test (not shown). *Credit:* Reprinted from Chandrasekaran et al. (2009). Copyright © 2009, with permission from Elsevier in respect to Chandrasekaran et al. (2009: Exp.1); *n* = 21.

Having established the FFRs of cABRs are influenced by auditory attention and long-term auditory experience, it is worth emphasizing that the cABRs generated in the rostral brainstem are not the automated readout of stimulus attributes in an informational vacuum. Rather, cABR generation is affected by expectancies derived from the immediate preceding context. An investigation of neural entrainment in children revealed such effects of acoustical context on cABR (Chandrasekaran et al., 2009). The notion was that a variable sequence of acoustically distinct monosyllables containing a /dɑ/ syllable prevents the preceding context from predictively enhancing the neural representation of the current stimulus /dɑ/. “Neural entrainment” using the context of a repeated /dɑ/ (Figures 6A,B) reflected such an enhancement. This neural entrainment enhanced the cABR second harmonic amplitude during the formant transition between consonantal and steady-state vowel portions of /dɑ/ (Figures 6C–E). The cortex could process a memory of the preceding context, leading to a top-down expectancy. Subcortical corticopetal-corticofugal loops attempt to meet that expectancy when encoding the current stimulation. The stronger such neural entrainment for the second harmonic in the formant transition, the better the speech-in-noise performance. Such neural entrainment of the cABR is thus functionally relevant for speech-in-noise performance.

This neural entrainment, enhancing the second harmonic during the formant transition, predicted speech-in-noise performance (Chandrasekaran et al., 2009) as assessed by the Hearing In Noise Test or HINT (Nilsson et al., 1994). The more faithful the cABR was to the auditory signal during the transition from the consonantal to the vowel portion, the better the speech-in-noise performance. This evidence concerning speech-in-noise performance of children coheres well with that from older adults. Anderson and Kraus (2010) compared two such adults, with near-identical audiograms (≤25 dB HL for audiometric frequencies from 125 to 8 kHz) to one another. The individual with poorer speech-in-noise peformance exhibited a weaker representation of the fundamental frequency and second harmonic in the FFR of the cABR. Comparing two groups of older adults who showed good and poor speech-in-noise performance, respectively, Anderson et al. (2011) found no significant audiometric difference (≤25 dB HL from 125 to 4 kHz), yet the difference in the FFR of the cABR was replicated. In those older adults, the presence of meaningless syntactic speech adversely affected the faithfulness of the cABR to a repeated /dɑ/. This influence of background speech noise was particularly strong in those showing poor speech-in-noise performance on the HINT: The higher the overall root mean square (RMS) of the cABR in quiet or noise, or the stronger the correlation of the cABR waveform to /dɑ/ in quiet and noise, the better the speech-in-noise performance on the HINT (Anderson et al., 2011).

Kindred to the neural entrainment of the cABR that predicted HINT performance (Chandrasekaran et al., 2009), Chandrasekaran et al. (2012) revealed that using repeated rather than changing stimuli augmented the FFR and reduced the cerebral blood flow in the IC: a repetition suppression effect. The processing of sound in the IC becomes more efficient when predictable. The fidelity of the FFR and the associated repetition suppression is particularly pronounced in those who have learned to process the sound well: e.g., English-speakers who rapidly learn new vocabulary based on the recognition of lexically meaningful tones, having acquired the mapping of distinct pitch patterns of one English pseudoword onto pictures of different objects. These findings could thus relate to the second language acquisition of tonal languages, such as Mandarin Chinese.

When sequences of natural stimuli such as speech exhibit an inherent acoustical variability with time, thus not promoting neural entrainment and repetition suppression, the nature of the filtering of the auditory information at the level of the rostral brainstem is thus arguably non-absolute. The IC shows increased bloodflow reflecting a less efficient processing of the stimulus and generates waveforms less faithful to the stimulus suggesting the filter is wide open to unpredicted stimuli. Accordingly, the experience-dependent corticofugal efferent influence on the rostral brainstem typically permits a selectivity for information promoted by top-down expectancies. Not only acoustical but also semantic and linguistic factors may influence expectancies. Those factors affect the ascendency of information in the auditory system from the IC upward. The influence of these top-down expectancies on corticopetal-corticofugal loops effectively operate as an early filter (Broadbent, 1958). The neural entrainment of facets of the cABR, FFR, or repetition suppression at the IC reflect the selectivity of that early filter, for instance, by affecting the perception of speech in noise. Yet the selectivity of that filtering is only near-absolute under conditions that promote neural entrainment or repetition suppression within the IC. These conditions are atypical in natural acoustically varying to-be-attended stimulation that is often in the presence of noise. The new early filter model offered here thus proposes that the early filter is not only affected by top-down experience-dependent selective attentional factors but also by neural entrainment. This assumption that neural entrainment affects the early filter is thus not as discrepant as Broadbent's (1958) early selection model was with the evidence supporting attenuation (Treisman, 1960, 1964a,b, 1969; Treisman and Riley, 1969) and late selection models (Gray and Wedderburn, 1960; Deutsch and Deutsch, 1963).

#### Interim summary

Top-down attentional as well as experience-dependent plasticity factors influence cABR generation. In support of an assumption of predictive selection by the early filter, this generation is also affected by the neural entrainment determined by the speech context. This neural entrainment affects the attention selectivity for speech in noise.

### TFS and age-dependent decline of temporal resolution

Having discussed how attention, expectancy, and prediction affect the sub-cortical representation of speech in the central auditory system, as well as speech-in-noise performance, we turn now to the representation of TFS. TFS is best understood by first considering how the auditory periphery analyses sound. The structure of the basilar membrane within the cochlea performs a Fourier-analysis-like function (von Békésy, 1960): The basilar membrane resolves a complex sound into component narrowband signals. In response to a sinusoidal stimulation, the basilar-membrane response takes the form of a traveling wave that shows a peak amplitude at a specific place on the basilar membrane, depending on the frequency of the stimulation. Due to the mechanical properties of the basilar membrane, the basal end responds most vigorously to high-frequency sounds and the apical end to low-frequency sounds. This tonotopically organized pattern of vibration is transduced by the inner hair cells. In the auditory nerve, each transduced component narrowband signal thus has a temporal envelope, an informational trace of the slow amplitude dynamics of the upper extremes of basilar membrane deflections of that narrowband waveform. This temporal envelope varies at lower frequency, slower than the higher frequency TFS information bounded within that envelope. This amplitude modulation envelope supplies cues to speech perception that are not only necessary but also sometimes alone sufficient for speech perception (Drullman et al., 1994a,b; Shannon et al., 1995). In quiet, slow-rate temporal-envelope cues (4–16 Hz) are especially important for speech identification (Drullman et al., 1994a) when higher frequency amplitude modulation envelope is present. Also in quiet, medium rate amplitude modulation envelope (2–128 Hz) is also important when lower frequency amplitude modulation envelope is absent (Drullman et al., 1994b). In the presence of interfering sounds, slow temporal-envelope cues (0.4–2 Hz) become important conveying prosody (Füllgrabe et al., 2009), as do high rate temporal-envelope cues (50–200 Hz) conveying fundamental frequency (Stone et al., 2009, 2010).

**Figure 7:**
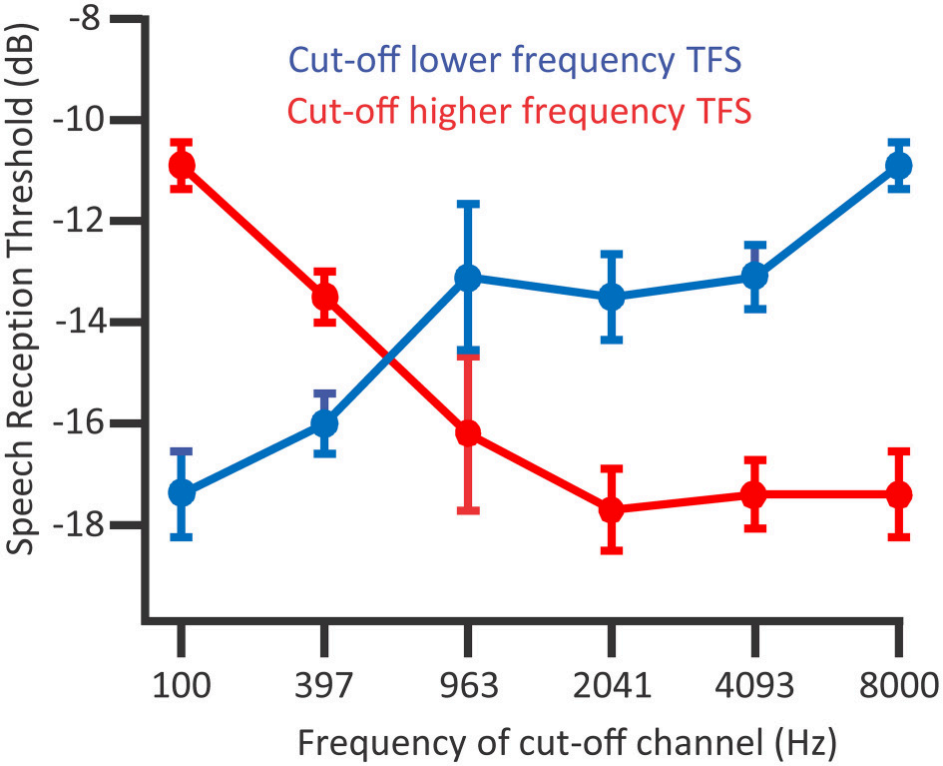
**The presence of TFS above 1500 Hz contributes to speech recognition thresholds in normal hearing listeners**. Considering the blue line, a procedure adding TFS between 8000 and 4093 Hz to tone-vocoded channels resulted in improved (lower) speech reception thresholds. Considering the red line, when lower frequency TFS is already available, after adding TFS between 963 and 2041 Hz that improved speech reception thresholds, there were no dramatic improvements from adding higher frequency TFS. Data points denote mean speech reception thresholds; error bars denote the standard error of the mean; *n* = 7. *Credit:* Reprinted with permission from Hopkins and Moore (2010). Copyright © 2010, Acoustic Society of America.

The temporal information bounded within this temporal-envelope is TFS, i.e., the fluctuations in amplitude close to the center frequency of a narrowband signal, which are higher in frequency than the amplitude modulation envelope. In tone-vocoded sound, narrow frequency bandwidths of sound—and in turn the resolved narrowband signal at the basilar membrane—“channels” have envelope information preserved yet the TFS replaced with a tonal sound amplitude-modulated by that envelope. Hopkins and Moore (2010) explored how incrementally replacing the content of tone-vocoded channels with the original speech channels improved speech recognition in noise. Listeners were between 19 and 24 years of age and audiometrically normal in the test ear. These listeners were sensitive to TFS as can be used in speech perception in noise: The speech reception thresholds of target signals containing partial TFS information improved when adding speech TFS information to the tone-vocoded sound (Figure 7; Hopkins and Moore, 2010). The TFS information improved thresholds in a procedure that incrementally replaced higher-and-higher frequency tone-vocoded channels with speech TFS (Figure 7, red line). TFS information also improved thresholds in a procedure replacing lower-and-lower frequency tone-vocoded channels of noise with speech TFS in the same bandwidth (Figure 7, blue line). Noteworthy is that TFS in higher frequency ranges aided speech recognition when no TFS was available in lower frequency ranges. In an analogous experiment, Hopkins and Moore (2010) also showed that speech TFS information is less useful to those with hearing impairment, albeit potentially confounded by the hearing-impaired participants being older (Moore et al., 2012; Füllgrabe, 2013; Füllgrabe et al., 2015).

Another study complements Hopkins and Moore's (2010) demonstration that TFS is important for speech identification in the presence of speech background sounds. Stone et al.'s (2011) experiments investigated the dynamic range of usable TFS information by comparing the addition of TFS information to the amplitude peaks of a vocoded speech signal by adding that TFS information to the valleys and troughs of this vocoded signal. Whether added to amplitude peaks or to troughs, TFS information improved identification of the target speech over a background talker: Adding target and background noise TFS information to a channel containing the corresponding temporal envelope information proved useful. This TFS information was useful for channel levels—relative to the RMS sound level of that channel—from about 10 dB below to 7 dB above that RMS sound level. However, the range of channel sound levels where TFS was useful depended on the relative levels of the target sound to the background masking talker: For an experimental condition in which background noise dominated the target more, adding TFS to peaks was more useful at channel sound levels further below the RMS sound level of the channel than in an experimental condition in which the background noise did not dominate the target as much. Further, adding TFS information to peaks when the background dominated more was more useful than adding TFS information to dips. Stone et al.'s (2011) results thus show that TFS information is not exclusively useful for listening in dips, but rather TFS also contributes to the segregation of target to-be-attended speech from the to-be-ignored background speech sound.

Having shown how processing TFS information is important to the recognition of speech in speech noise, we turn to how the subcortical processing of TFS is relevant to one of the outstanding unresolved conundrums of cognitive hearing science. This conundrum is that of isolating the age-related decline in temporal processing that is caused by effects of peripheral hearing loss on the auditory nerve and central auditory system from age-related declines that are unconnected to audiometric loss. Presbycusis, age-related sloping loss, may drive a progressive deafferentation of unstimulated neurons spreading upward in the ascending auditory system, which ultimately results in chronic cognitive change according to the sensory deprivation hypothesis. Hearing-impaired listeners can experience supra-threshold auditory processing deficits, characterized by distorted processing of audible speech cues. Peripheral damage to outer hair cells and reductions in peripheral compression and frequency selectivity contribute to these deficits, as does a reduced access to TFS information in the speech waveform, leading to this distortion (Summers et al., 2013). However, this impairment of TFS processing, which affects distortion, is not necessarily always a direct or indirect consequence of peripheral damage.

There is evidence for an independent age-related decline in temporal resolution as reflected by the action of the rostral brainstem of the central auditory system (Marmel et al., 2013)^2^. Marmel et al. investigated an audiometrically heterogeneous population of adults with a wide age range (Supplementary Figure 1A). Participants with thresholds greater than 20 dB HL had a sensorineural loss. To investigate inter-individual variability in temporal resolution at the level of the rostral brainstem of the central auditory system, Marmel et al. used an FFR synchronization index. This index comprised of the cross-correlation of FFR to the stimulus and also comprised of the signal-to-noise ratio of the FFR. Such an index thus tracked how faithful the FFR was to the acoustical stimulus. This FFR synchronization index decreased with age in a manner reflecting a poorer temporal resolution at the level of the rostral brainstem, which is associated with higher frequency difference limens. These higher limens reflected poorer pitch discrimination abilities. A tendency for sloping loss to be more severe in elder participants was confirmed (Supplementary Figure 1), yet at 500 Hz, hearing thresholds did not correlate significantly with age (Supplementary Figure 1C). Marmel et al. presented stimuli in this frequency range when measuring absolute auditory thresholds, frequency difference limens, and FFR synchronization. The influence of age on FFR synchronization in this frequency range, without a significant influence of age on hearing level, thus strains any assumption that a peripheral presbycusis could be the sole cause of this effect of age on the processing of sound by the central auditory system (though see Footnote 2). Further, this FFR synchronization was not associated with absolute auditory thresholds. The point is that there was an age-related decline in temporal resolution arguably at the level of the rostral brainstem that was associated with impairments in pitch discrimination abilities. Pitch discrimination abilities appeared to hinge both on absolute auditory thresholds and on the FFR synchronization index. However, FFR synchronization yet not absolute auditory threshold was affected by age. It is tenable that auditory absolute threshold could affect the place-coding of auditory information, in turn affecting pitch discrimination. Equally, absolute auditory threshold could affect the coding of auditory information that is not phase-locked. However, the firing of neurons conveying that auditory information would have to be asynchronous. The upshot of Marmel et al.'s (2013) findings is that there is an age-related functionally relevant decline in auditory temporal resolution at the level of the rostral brainstem. This decline is arguably independent of audiometric hearing loss, which though affecting frequency discrimination, was not affected by age within the frequency ranges investigated.

The question that still remains is whether aging of the auditory nerves and central auditory system alone drives this functionally relevant decline of temporal resolution arguably at the level of the rostral brainstem, as indexed by FFR synchronization. Such aging could relate to a decline of inhibitory GABAergic (Caspary et al., 2008; Anderson et al., 2011) or cholinergic (Zubieta et al., 2001) systems of neurotransmission. Such systems involve respectively, γ-aminobutyric acid or acetylcholine. A decline in temporal processing may limit the speed of acoustical fluctuations that the (auditory nerve and, in turn the) central auditory system can follow. Such a decline thus renders it impossible for the central auditory system to represent high frequencies using the rate facet of a place-rate code, which affect the IC's generation of the FFR.

#### Interim summary

There is an age-related decline in supra-threshold auditory processing, which Marmel et al. (2013) revealed as independent of audiometric hearing loss (Marmel et al., 2013). This age-related decline occurs alongside a decline in the temporal resolution of the FFR, which arguably the rostral brainstem generates. This age-related decline in temporal resolution could also impair speech recognition in noise (Hopkins and Moore, 2010). There is a comparable age-related decline in TFS sensitivity, which even occurs in audiometrically normal adults (Füllgrabe, 2013; Füllgrabe et al., 2015). However, peripheral hearing loss could also drive a decline in the processing of sounds in the auditory nerves and central auditory system. This loss is either measurable in the audiogram, or is “hidden” (Schaette and McAlpine, 2011; Plack et al., 2014).

### Neuroplastic changes to accommodate high-frequency audiometric loss

**Figure 8:**
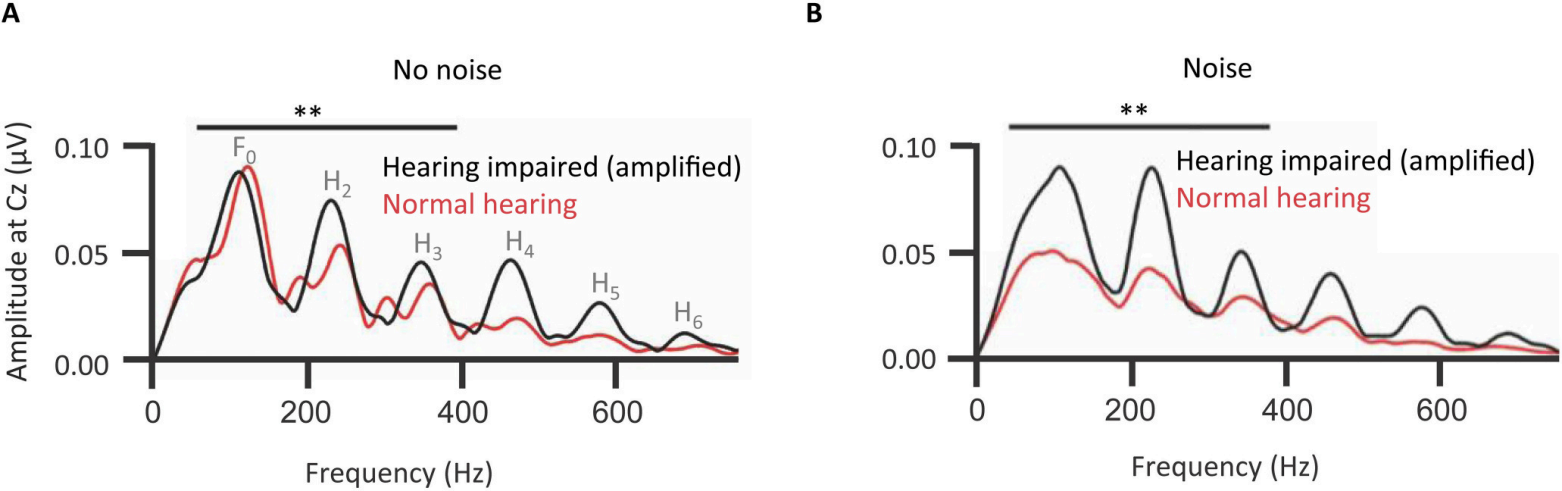
**Long-term neuroplastic changes accommodate peripheral sensorineural hearing loss: Effects of hearing loss on the amplitude spectra of the envelope FFR of grand-averaged cABR representing the fundamental and lower harmonics of the vowel portion of a /dɑ/ stimulus under acoustical background conditions of noise or no noise**. The fundamental and lower harmonics (F0 to H3) were together represented **(A)** significantly more strongly (denoted by ^**^) in elderly individuals with mild-to-moderate sloping hearing loss (*n* = 15), than in elderly controls without such a loss (*n* = 15). Background noise (pink noise of signal-noise ratio 10 dB) exacerbated this effect **(B)**. There was no hearing impairment-associated effect in higher harmonics. Arguably, hearing-impaired participants have learned to rely on lower-frequency speech cues, particularly in noise. With the same hearing-impaired listeners without amplification the pattern of significance replicated, though these effects were slightly weaker without amplification (not shown). *Credit*: Reprinted with permission from Anderson et al. (2013). Copyright © 2013, Acoustic Society of America.

A hypothesis is that the decline of temporal resolution of the central auditory system, indexed by the FFR, comes from long-term neuroplastic change to accommodate the loss of audiometric sensitivity, especially in the high-frequency range. Older adults with mild-to-moderate hearing impairment show FFRs of the cABR with an, at first counterintuitive, higher amplitude fundamental and lower harmonics than normal-hearing controls (Figure 8; Anderson et al., 2013). One explanation is the higher amplitude of the FFR in hearing-impaired listeners might be due to a larger effective modulation depth in those listeners caused by the reduction or abolition of cochlear compression (Füllgrabe et al., 2003; Oxenham and Bacon, 2003).

However, such results are also germane to another theory (Woods and Yund, 2007) that sensorineural impairment leads to a remapping from the auditory cortex—with impoverished output to high frequency cues—to the auditory association cortex. Accordingly, that remapping, to compensate, takes the low frequency cues still available for phoneme recognition and amplifies those cues within the central auditory system (Woods and Yund, 2007). Whether occurring between the auditory cortex and auditory association cortex, or between other structures of the auditory system, this remapping has consequences. Anderson et al.'s (2013) data relate to such a remapping. Those consequences alter the generation of the FFR in the rostral brainstem. Anderson et al.'s (2013) analyses of higher harmonics Aiken and Picton (2008) revealed no corresponding upregulation of high frequency cues.

Further, Anderson et al.'s analyses revealed that whether the stimuli were unamplified or amplified, using the NAL-R fitting formula (Byrne and Dillon, 1986), there was a bias in persons with mild-to-moderate hearing loss toward a stronger upregulation of lower rather than high frequency components in noise. Indeed, this bias for upregulating high frequency components was even stronger when amplified. If this bias were due to peripheral factors alone, such as a reduction in cochlear compression, then, if there were no long-term consequent neuroplastic changes, we would predict amplification would attenuate that bias. Anderson et al.'s analyses revealed the reverse of that prediction: amplification enhanced this bias. Accordingly, while peripheral factors such as declining cochlear compression would affect the FFR, long-term neuroplastic changes also take place that affect the FFR. Amplification with the NAL-R formula used did not remediate these changes.

Anderson et al.'s (2013) FFR findings from older adults with mild-to-moderate hearing impairment cohere well with evidence of a slightly different sort. Upon receiving an aid that amplifies high frequency cues, hearing aid users who have had unaided high frequency hearing loss for many years, can hear the amplified sound as distorted (Woods and Yund, 2007; Galster et al., 2011). Reasons, which are not necessarily mutually exclusive, could include regions of dead cochlea (Vickers et al., 2001; Mackersie et al., 2004; Moore, 2004; Preminger et al., 2005; Aazh and Moore, 2007; Vinay and Moore, 2007; Zhang et al., 2014). However, other reasons could include the long-term plasticity of the auditory nerves or central auditory system attempting to make the best use of lower frequency information from a damaged periphery. The low frequency sound can also seem too loud: “hypercusis.” Here the notion is that the encoded low frequency cues swamp high frequency perceptual cues. This problem is even more apparent in the FFR under conditions of background noise (Anderson et al., 2013). As Galster et al. (2011) note, “the inability to restore audibility of high-frequency speech and the possible contraindication for the restoration of high-frequency speech are established conundrums of hearing care.” This neuroplastic change is, at least in part, reversible. Training programs improve the aided perception of word-initial phonemes for those who have become accustomed to high frequency loss (Woods and Yund, 2007); people who presumably have partially functional basal cochlear regions. The neuroplastic changes, which adapt to hearing loss and seem to implicate the rostral brainstem, thus seem, on the whole, to be reversible. These neuroplastic changes are reversible even in later life and even after extensive hearing aid use. At first glance, such a finding would cohere well with the notion of neuroplastic recovery from neuroplastic long-term changes that accommodate peripheral hearing loss. However, it is worth considering that GABA units can increase in the auditory cortex due to training (Guo et al., 2012). Accordingly, systems of neurotransmission could have aged affecting the temporal resolution of the central auditory system to a point that is not normal. Those systems of neurotransmission could be subject to recovery due to training. While training was effective for nearly all individuals, there were factors affecting the inter-individual differences in the efficacy of training (Stecker et al., 2006). The distortion and annoyance issues associated with receiving an aid after becoming accustomed to sensorineural hearing impairment also concern signal processing techniques. These techniques map information in the high frequency components in the to-be-amplified sound onto lower frequency regions of cochlea (Galster et al., 2011). Approaches include frequency compression (Glista et al., 2009) and frequency transposition (Füllgrabe et al., 2010).

Such a signal processing approach might be more advisable than training when the majority of high frequency (basal) regions of cochlea are dead—the relevant afferents of the eighth cranial nerve have atrophied. At first, it is hard to imagine how such individuals could benefit from a training in listening to high frequency information: If a region of cochlea is dead, there is no sound transduction at the characteristic frequencies of the inner hair cells of that region. However, if sounds are loud, a frequency component produces a broader excitation pattern across auditory nerve fibers. With loud enough frequency components, regions of live inner hair cells neighboring dead cochlear regions, would thus be able to transduce some high-frequency information: “off-frequency listening” (Westergaard, 2004). Foreseeable is that training these persons to use information from off-frequency listening might have some benefit with very high levels of amplification. For persons with extensive dead basal cochlear regions, a prediction is that such training is not as effective as the suggested signal processing approaches.

**Figure 9:**
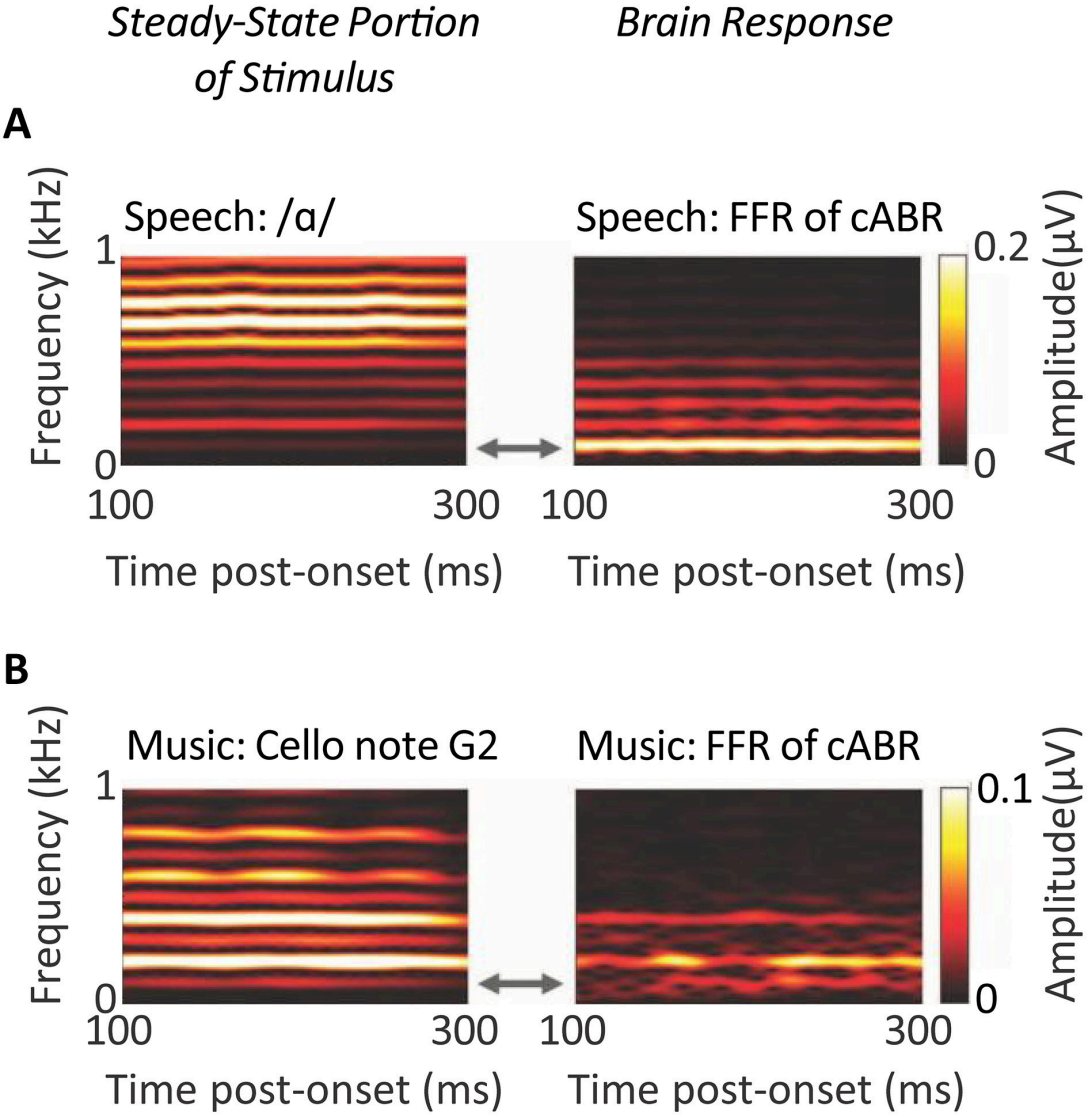
**Evidence for the low-pass properties of the auditory brainstem**. Spectrograms of steady-state portions of speech **(A)** and non-speech **(B)** stimuli (left-hand panels) reflect the same fundamental frequency of 100 Hz, but a different harmonic structure. The corresponding spectrograms of the Frequency Following Responses of the cABR (right-hand panels), reveal that FFR follows the fundamental and lower harmonics more strongly than higher frequency harmonics; *n* = 29. *Credits:* Adapted with permission from Musacchia et al. (2007). Copyright © 2007 National Academy of Sciences, U.S.A., after Skoe and Kraus (2010). Adapted with permission from Skoe and Kraus (2010). Promotional and commercial use of the material in print, digital or mobile device format is prohibited without the permission from the publisher Wolters Kluwer Health. Please contact healthpermissions@wolterskluwer.com for further information.

Anderson et al.'s (2013) analyses offer intriguing biomarkers to evaluate for specifity in predicting such treatment's outcomes. These analyses were geared to investigating both lower frequency components and higher frequency components of the FFR of the cABR. As such these analyses revealed low frequency cues swamp higher frequency cues following neuroplastic changes that accommodate sensorineural loss. By contrast to these analyses, the representation of the stimulus classically apparent in the cABR, is relatively abstracted from the TFS at the level of the rostral brainstem. Whether the FFR of the cABR was responsive to the steady-state segment of a vowel or the steady-state sound of a cello, that FFR represented the fundamental and lower harmonics more strongly than the higher harmonics: As depicted in Figure 9, such lower frequency components were more strongly represented even when higher harmonics are of a higher intensity, as attributable to the low-pass characteristics of brainstem phase-locking (Musacchia et al., 2007; Skoe and Kraus, 2010).

#### Interim summary

Audiometric hearing loss could drive a decline of temporal resolution in the central auditory system. This age-related decline could be a long-term adaptation to higher frequency loss at the periphery. However, Füllgrabe et al. (2015) have shown an age-related decline of temporal resolution in audiometrically normal individuals, who are audiometrically matched across age groups. This decline thus arguably occurs in the central auditory system. This finding would thus indicate that audiometric hearing loss does not drive all such decline. However, this assertion comes with a caveat that there may be hidden loss (Schaette and McAlpine, 2011; Plack et al., 2014; Kujawa and Liberman, 2015) that is age-related. Accordingly, that hidden loss does not affect the audiogram but still drives this decline thus affecting the central auditory system. The cABR can reflect neuroplastic changes in response to peripheral sensorineural loss upregulating the relative representation of lower rather than high frequency components arguably at the rostral brainstem. This upregulation occurs in a manner exacerbated by noise and by amplification, as could relate to distortion, hypercusis, and annoyance issues. The cABR is thus an intriguing biomarker that could have specificity informing the approach to treatment. Stimulus transduction artifact-free cABRs can now be recorded through hearing aids (Bellier et al., 2015). It remains to be determined how well such cABR attributes—including noise sensitivity and the extent of adaptation to lower frequency components—predict the outcomes of fitting. This fitting concerns signal processing, directional microphones, binaural care, and choice of noise reduction schemes. Also to-be-determined is how well such cABR attributes predict the benefit from behavioral interventions such as perceptual training (Woods and Yund, 2007).

### From the limits on phase-locking in the inferior colliculus to top-down neural entrainment during speech perception

We have seen that prolonged hearing impairment has consequences for the generation of FFR of the cABR, which typically reflects low frequency sound components. The temporal envelope information in a narrowband signal is definitively lower in frequency than the TFS information bound within that envelope. Narrowband signals with a lower center frequency, are, however more dominated by temporal envelope information than narrowband signals with a higher center frequency. Much speech TFS information is transmitted through those higher center frequency narrowband signals. The question remains for neuroscience as to how TFS is re-coded prior to the rostral brainstem. Spectral FFRs are known to represent harmonics of acoustical information as high as 1500 Hz (Aiken and Picton, 2008). Yet, a processing of TFS above 1500 Hz contributed to speech target recognition in speech background noise (Hopkins and Moore, 2010). The frequency components of that TFS over 1500 Hz are thus somehow processed by the brain. Such temporal information is available at the level of the cochlear nucleus (Palmer and Russell, 1986; Winter and Palmer, 1990). Skoe and Kraus (2010) postulate that a place code facet of a rate-place code (Rhode and Greenberg, 1994) recodes information about higher frequencies. Such a place code could be supported by a form of tonotopy within the IC (e.g., Malmierca et al., 2008). Indeed, Harris et al. (1997) support this notion of a rate-place code with multi-unit recordings from gerbil IC. Frequencies below 1000 Hz activated a broad phase-locked population. Higher frequencies induced activation of a more focal population without phase-locked firing of the constituent neuronal elements. The spectral FFR is thus more strongly affected by lower frequencies.

**Figure 10:**
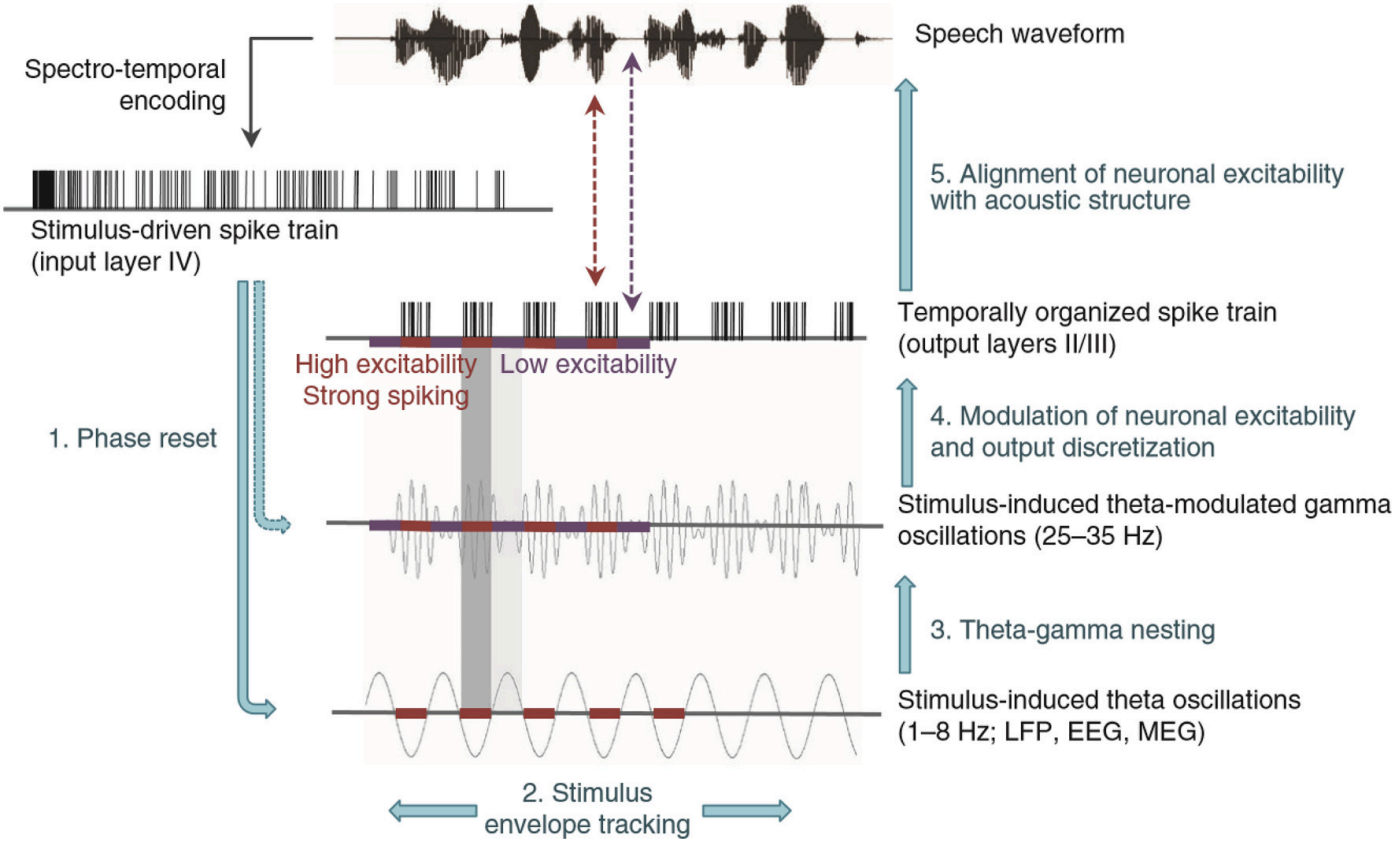
**Giraud and Poeppel's theory of speech perception: Cortical theta and gamma oscillations parse connected speech**. This five-step theory assumes a high-resolution spectro-temporal representation of speech in primary auditory cortex. This representation cannot rely only on the phase-locked synchronous firing of neurons that generate the FFR of the cABR up to 1500 Hz. Rather, a place-rate code must constitute such a(n asynchronous) cortical input. Thus, a typical spike train inputs deep layer IV cortical neurons, which phase-lock to speech amplitude modulations. Response onset elicits a reset of theta oscillations in superficial layers II and III (step 1)—the output of the auditory cortex. After this reset, theta oscillations follow the speech envelope (step 2). In turn, that theta reset causes a brief pause of gamma activity and a subsequent reset of gamma oscillations. The coupling of theta and gamma generators becomes both stronger and “nested” such that the phase of speech envelope following theta oscillations controls the phase and power of the gamma oscillations (step 3). This gamma power controls the excitability of neurons generating the feedforward signal from primary auditory cortex to higher order areas (step 4). This neuronal excitability phase aligns to speech modulations (step 5). We postulate such cortical modulations of neuronal firing by neuronal oscillations parse auditory input and serve as a context that corticofugally influences subcortical neural entrainment of corticopetal-corticofugal loops. Accordingly, linguistic factors can promote the perception of speech in noise in a top-down manner. *Credit:* Reprinted by permission from Macmillan Publishers Ltd: NATURE NEUROSCIENCE (Giraud and Poeppel, 2012), Copyright © 2012.

Turning from these evoked responses to neuronal oscillations, a recent model of how cortical theta (1–8 Hz) and gamma (25–35 Hz) oscillations process speech assumes a high-resolution spectrotemporal representation of speech in the primary auditory cortex (Figure 10; Giraud and Poeppel, 2012). This representation enters input layer IV upon which operations are performed to code speech into the theta- and gamma-band, albeit a representation encoded in a neuronal spike train. At first blush, the assumption of such a representation contrasts with the upper limit of phase-locking in the FFR. This limit is known to drop from 3.5 kHz in the guinea pig auditory nerve to 2–3 kHz in the cochlear nucleus (Palmer and Russell, 1986; Winter and Palmer, 1990) down to 1000 Hz in the central nucleus of the guinea pig's IC, right down to 250 Hz in auditory cortex (Wallace et al., 2002, 2005). This cortical limit is likely an over-estimate in non-human primates (Steinschneider et al., 1980, 2008), perhaps even humans.

We postulate an asynchronous recoding at the input to the auditory cortex serves as a high-resolution spectrotemporal representation of speech in a spike train within the primary auditory cortex. This cortical representation is strongly reliant on the place facet of a rate-place code, particularly for higher frequencies. Such representation is a spike-coded high-resolution representation that interacts with a gamma-band representation of spectrotemporal information in the speech bandwidth compressed into a lower frequency range (25–35 Hz). This gamma-band representation interacts with a slow stimulus-locked theta-band representation (1–8 Hz), further refining the spike coding of speech information for cortical processing of meaningful utterances (Giraud and Poeppel, 2012). Regions of auditory cortex show high measurements of GABA+; GABA+ levels correlating positively with language skill on the CELF4 (Gaetz et al., 2014), such that autistic children exhibit decreased GABA+ and auditory gamma-band (30–50 Hz) responses to auditory pure tones (Gandal et al., 2010; see also Port et al., 2015). A possibility is that gamma power may thus modulate the auditory cortical excitability to refine the spike coding of speech information for cortical processing of meaningful utterances. The corticofugal influence of such cortical modulations by neuronal oscillations are postulated here to serve as a possible basis of subcortical neural entrainment: Rather than the repeated speech context entraining the processing of the speech by the rostral brainstem, the parsing of the utterance entrains that processing.

#### Interim summary

The cABR reflects, at least in part, the phase-locked responding of a wide neuronal population within the IC tuned to the fundamental and lower harmonics of the acoustical stimulus. This phase-locking of a wide population breaks down in exchange for more focal populations that are tonotopic to higher frequencies and do not contribute strongly to the cABR. Higher frequency components of greater than 250 Hz cannot be cortically represented in a phase-locked manner, though such components are perceptually relevant to TFS perception that can improve speech-in-noise perception. Rather, a high fidelity spatiotemporal representation arguably enters the primary auditory cortex as a neuronal spike train. That spike train representation interacts with the lower frequency range compression of speech sound of the low gamma-band alongside a stimulus-locked representation of the sound in the lower theta band. These interactions of the spike trains with these neuronal oscillations in auditory cortex, we posit, not only refine the syntactic and semantic processing of the speech, but also have top-down corticofugal influences. Germane is that a memory for a repeated rather than a variable context enhances the subcortical representation of incoming stimulation in a manner that indexes speech-in-noise perception (Chandrasekaran et al., 2009). Just as that enhancement could rely on top-down prediction, these interactions of the spike train with auditory cortical neuronal oscillations could control corticopetal-corticofugal loops to promote the subcortical processing of semantically and syntactically predictable utterances. Those neuronal oscillations in the cortex could corticofugally modulate subcortical neural entrainment, such that top-down (semanto-syntactic) speech context can affect early filtering. Just as it is assumed that the new early filter operates by predictive selection on the basis of acoustical context, there is also scope for semanto-syntactic context to influence that predictive selectivity.

### Section summary

The ascending auditory system contains a series of relay stations that generate the ABR to sounds. This ascending auditory system is part of multiple corticopetal-corticofugal loops that can dynamically adapt to filter information selectively on the basis of top-down control by higher structures. The manipulation and temporary storage of contextual information in the prefrontal cortices affects how the cortical cholinergic system controls those loops in a top-down manner. The connectivity of the IC serves as a hub of this early filter at the confluence of the bottom-up processing of the ascending auditory system, binaural interactions, and the top-down controlled predictions from the descending auditory system. There is thus the connectivity to support attentional modulations of ABRs, as occurs under conditions including loud noise. By contrast to cortically generated ALLRs such as the N1 (e.g., Butler, 1973; Campbell et al., 2003, 2005, 2007; Campbell and Neuvonen, 2007), such ABRs are relatively unaffected by refractoriness (Picton et al., 1992; Valderrama et al., 2014). Accordingly, populations of ABR-generating neurons are not particularly susceptible to refractoriness. Thus, an ongoing sound leads to an ongoing cABR to acoustical landmarks within that sound. Top-down contextual factors can influence the generation of that cABR. Indeed, we would argue that stimulus and linguistic context affect the subcortical representation of speech in a top-down manner.

The ability to process TFS information is not apparent in the cABR that typically has low-pass characteristics. There is an age-related decline in temporal resolution, even when there is no audiometric evidence for sensory decline. Processing TFS information is important for speech perception in noise. The sensory deprivation and information degradation hypotheses (Schneider and Pichora-Fuller, 2000) for this decline in temporal resolution still cannot be out-ruled: Hidden loss, which is immeasurable with an audiogram, might still drive that decline. Similarly, there could be a sensory processing of that TFS, which is intrinsically intertwined with a cognitive processing of TFS. A decline in this cognitive processing could cause a decline in the supra-threshold sensory processing of TFS, as postulated by Schneider and Pichora-Fuller's cognitive load on perception hypothesis. With a /dɑ/ stimulus, the cABR typically neglects the higher harmonics dominated by TFS rather reflecting the brain's ongoing response to the fundamental and lower harmonics. Nevertheless, the cABR offers a promising biomarker with respect to speech-in-noise perception. Whether the addition of cABR to an audiologist's diagnostic battery would improve the specificity of treatment outcome remains undetermined. A recent investigation, to which we now turn, used an approach to cABR to glean higher harmonics that bear considerable TFS information—harmonics that could offer insights into the nature of an individual's speech perception under adverse conditions such as noise or reverberation.

## Reverberation and processing of TFS by IC

Fujihira and Shiraishi (2015) investigated the FFR of the cABR of elderly individuals (61–73 years) with age-normal hearing. Pure-tone audiograms revealed listener's overall mild hearing loss to be age-normal: That loss was in no case strongly asymmetric and latencies of a discernable click-evoked wave V were normal for each participant. Pure tone averages (500–4000 Hz) revealed bilateral losses less than or equal to 30 dB HL, while thresholds at 8000 Hz were less than 50 dB HL. To obtain cABRs, each participant heard a series of /dɑ/ speech sounds in rapid succession—instances of the original acoustical waveform were interspersed with an inverted version that was 180° out-of-phase with the original.

EEG epochs were time-locked to the onset of each acoustical waveform. There were an equal number of epochs free of bioelectric artifacts selected containing responses to the original and the inverted waveform. Two separate sets of epochs were binned according to stimulus type. From these sets of epochs, Fujihira and Shiraishi derived two different kinds of FFRs of the cABR to /dɑ/. Each such response followed either the spectral frequency of the stimulus or the frequency of that stimulus's envelope. Fujihira and Shiraishi then used these sets of epochs in two different forms of analysis (Aiken and Picton, 2008) with the purpose of isolating: (i) the envelope FFR that phase-locks to the periodicity envelope using what is termed the ADD method; (ii) the spectral FFR that phase-locks to resolved harmonic components of the acoustical signal thus containing some of TFS resolved by the auditory periphery using what is termed the SUB method.

**Figure 11:**
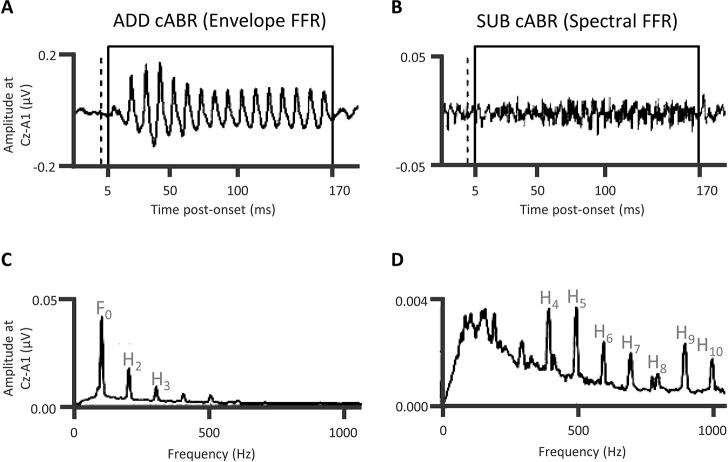
**A higher frequency TFS is represented in cABRs than previously thought**. Grand-averaged “envelope FFR” and “spectral FFR” of elderly listeners with intact hearing derived respectively with ADD **(A)** and SUB **(B)**. The SUB waveform **(B)** arguably exhibits intact TFS. The dotted line denotes the onset of /dɑ/, boxing the time-lagged period when the brain is responsive to the sound, time-lagged from the onset by the time stimulus information takes to reach the rostral brainstem. Fujihira and Shiraishi (2015) separately derived the corresponding amplitude spectra using ADD **(C)** and SUB **(D)** methods of Aiken and Picton (2008). *Credit:* Reprinted from Fujihira and Shiraishi (2015). Copyright © 2015, with permission from Elsevier; *n* = 30.

On the one hand, the individual ADD cABR came from EEG epoch waveforms collapsing across original and inverted /dɑ/ epochs, in the classical manner (Skoe and Kraus, 2010). This approach reduced the contamination in the recordings from the cochlear microphonic and from any stimulus transduction artifact (Aiken and Picton, 2008; Campbell et al., 2012). This ADD cABR (Figure 11A) reflected the time-lagged course of the stimulus envelope abstracted away from the TFS inclusive of higher harmonics of the acoustical waveform. This ADD cABR also represented well the fundamental frequency and lower harmonics (Figure 11C).

On the other hand, for the individual SUB cABR, EEG epoch waveforms in response to inverted /dɑ/ epochs were subtracted from original /dɑ/ epochs and divided by the total number of responses (for a similar approach, see also Anderson et al., 2013). In comparison to the ADD cABR phase-locked to the periodicity envelope, this low amplitude polarity-sensitive SUB cABR (Figure 11B) neither represented well the fundamental frequency, lower harmonics, nor the stimulus envelope. Instead, the SUB cABR reflected the TFS and higher harmonics of the acoustical waveform (Figure 11D). This SUB cABR mostly reflected the spectral FFR. While the cochlear microphonic could have contributed to this SUB cABR, the precedent is that the cochlear microphonic is not influential (Aiken and Picton, 2008): Only one harmonic was significant with masking that rendered a speech stimulus inaudible (Aiken and Picton, 2008: Exp.2). The resulting response arguably reflected the cochlear microphonic in the absence of either a brainstem-generated polarity-sensitive neuronal frequency following response (Chimento and Schreiner, 1990) or an auditory nerve-generated neurophonic. Aiken and Picton revealed multiple significant other harmonics not attributable to the cochlear microphonic. There were no such frequencies higher than 1500 Hz in the spectral FFR, despite there being harmonics higher than 1500 Hz in the acoustical stimulus. Further, Fujihira and Shiraishi's acoustical stimulation via tubes (Killion, 1984), with a transducer distant from the participant and EEG recordings, precluded substantial contamination of the SUB cABR stimulation transduction artifact.

Rather, the polarity sensitivity of the SUB cABR arguably relates to the asymmetry of the speech signal's envelope alongside the brainstem's reflection of slight polarity differences in phase-locked activity to periodicity envelope encoded from several regions of cochlea. The SUB cABR thus conveys a complex sum of TFS information from multiple narrow frequency bands resolved at the cochlea. With a /dɑ/ stimulus, envelope dominates the resolved low frequency bands apparent in the ADD cABR. The influence of high frequency TFS content on that periodicity envelope arguably dominates the resolved high frequency bands apparent in the SUB cABR.

Fujihira and Shiraishi tested the association between aspects of this SUB and the ADD cABR with word recognition of isolated familiar words under anechoic conditions and under reverberatory conditions at multiple reverberation times (0.5, 1.0, and 1.5 s): Overall, the longer that reverberation time, the less intelligible the speech. Neither SUB nor ADD cABR responses predict word recognition under anechoic conditions—performance being at ceiling. However, aspects of the SUB yet not the ADD cABR responses predicted word recognition of single words in isolation under reverberatory conditions.

That is, correlation matrices of amplitudes of components of the discrete Fourier Transform of SUB and ADD cABRs with word recognition performance showed that the amplitude of the ADD cABR did not predict this performance under reverberation. Contrastingly, the amplitude of the high harmonics in the SUB cABR did: The amplitude of SUB cABR harmonics at around 400, 500, 800, 900, and 1000 Hz correlated positively with word recognition performance for at least one reverberation time; the amplitude of the harmonic around 500 Hz correlating positively with word recognition under all reverberatory conditions. In other experiments, EFRs, phase-locked to the fundamental, have shown a reliable polarity-sensitivity in a subset of individuals (Aiken and Purcell, 2013; Easwar et al., 2015) albeit a polarity-sensivity that was not significant on a group level. The contribution of such individual differences in the generation of the polarity-sensitivity of the EFR could not account for such correlations of amplitudes of higher harmonics in the SUB cABR with word recognition. Rather, Fujihira and Shiraishi thus arguably showed that the phase-locked brainstem coding of TFS is critical to word recognition under adverse conditions of reverberation. Fujihira and Shiraishi conjecture that TFS is present in the higher harmonics of the spectral FFR of the cABR. Appealing as this explanation is, however, it remains to be discerned what TFS narrowband signal components bear individually within the frequency range of 400-1000 Hz at the level of the IC. It is further worth considering that perceptual compensation (Watkins and Raimond, 2013) for effects of reverberation were possible in the word recognition task, given reverberation time was consistent within blocks. Accordingly, the TFS arguably manifest in the spectral FFR of the SUB cABR to /dɑ/ sounds in quiet could index the influence of top-down expectancy of a repeated /dɑ/ on the rostral brainstem representation of that /dɑ/. This capacity for top-down expectancies to influence subcortical processing could have also affected perceptual compensation. Accordingly, this perceptual compensation operates by selecting the crucial TFS information required for speech-in-noise performance.

The contralateral presence of speech-shaped noise in Fujihira and Shiraishi's tests of word recognition could promote the binaural interaction of information from to-be-ignored masking noise. This interaction arguably invoked similar descending corticofugal, effortful, mechanisms of top-down selective attention affecting processing at the level of the rostral brainstem (Maison et al., 2001; Ikeda et al., 2008, 2013). Further, preceding context could affect the spectral FFR of cABRs (Chandrasekaran et al., 2009, 2012). Use of that context in spectral FFRs could relate to an ability for perceptual compensation (Watkins and Raimond, 2013), much as the greater influence of a repeated relative to a variable syllabic context on envelope FFRs (Figure 6) is associated with speech-in-noise performance (Chandrasekaran et al., 2009). Thus, the sound of prior heard speech context gave rise to a mental model of the tacit knowledge of the room acoustics. Accordingly, participants' brains used that model to influence the effect of reverberation on speech perception. Using such context required the formation of a model of the stimulus or the room acoustics, a model retained in memory to influence perceptual performance.

### Section summary

One thousand five hundred Hertz represents the limits of phase-locking at the auditory brainstem (Aiken and Picton, 2008). The attenuation of functionally relevant information in higher frequency components (>400 Hz) in contralateral noise is not as a strong an attenuation as with the lower frequency components revealed by the traditional ADD technique. Fujihira and Shiraishi employed the SUB technique to derive spectral FFRs.

In assessment, those with intact hearing, and to a lesser extent those with mild-to-moderate hearing loss, make use of TFS in frequency ranges greater than 1500 Hz for speech perception: Some form of re-coding of TFS seems inevitable to give rise to modulations of gamma- and theta-bands oscillations at the level of the cortex. Just as the representation of (semanto-syntactic) speech context parsed at the level of the cortex could affect the subcortical representation of speech at the level of the rostral brainstem, so could the representation of the room acoustics also parsed at the level of the cortex. These findings cohere with the predictive selection assumption: It is postulated that such top-down expectancies from context corticofugally modulate the rostral brainstem processing of phase-locked speech information in corticopetal-corticofugal loops. Accordingly, those expectancies critically influence the speech word perception and in turn word recognition in a context of adverse reverberatory conditions. For such a context to influence the rostral brainstem processing requires a WM function for complex span tasks. This function is the retention of that context in memory storage whilst processing the dual task of listening-to or recognizing the speech. It is to the influence of memory abilities on ABR generation to which we now turn, in which the findings of a recent investigation are germane.

## Auditory brainstem responses, working memory, and speech in noise

Humans have the ability of perceptual compensation, i.e., using prior context to help perceive speech correctly, an ability that relies on memory. For instance, knowledge of the room acoustics from immediate prior speech sound context reduces the adverse effects of reverberation on speech perception (Watkins and Raimond, 2013). When listening, the brain thus holds a mental model of the room's acoustics in (working) memory. The brain uses that model in a top-down prediction to select the perceptual representation of the current utterance, so as to support speech perception. A hypothesis is that some (working) memory function of the brain interacts with an early stage of processing in the brainstem to support that predictive selection. This hypothesis is of interest because the extent to which the brain can use contextual information held in (working) memory for top-down predictive selection at the subcortical level of the brainstem, in turn, would influence speech-in-noise perception. The result of a study of ABRs to ignored sounds under different conditions of load, for people with different working memory capacities evaluates this hypothesis.

**Figure 12:**
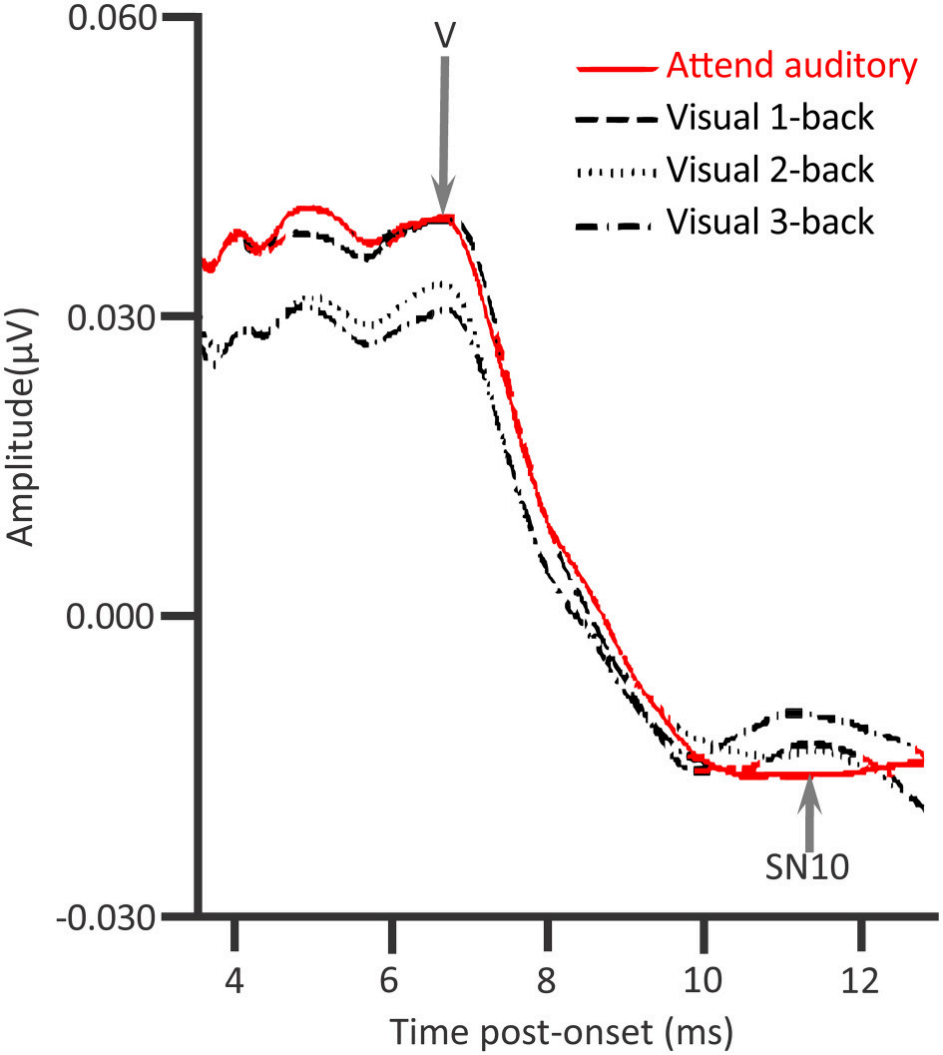
**Concurrent memory load and working memory capacity affect ABRs**. The amplitude of Wave V of click-elicited auditory brainstem responses decreases with visual(-verbal) memory load on working memory capacity (WMC). Note SN10 mirrored performance rather better than Wave V: SN10 was reduced by the load of the 3-back relative to the 2-back, indicating performance-limiting processes that are possibly cortical, in turn leading to more error-prone *n*-back performance (1-back = 2-back < 3-back). On the high-load 3-back task, the higher an individual's WMC, the lower the amplitude of Wave V. Working memory capacity limits a top-down system that corticofugally suppresses to-be-ignored sound. The cortical cholinergic system is such a top-down system. The red line depicts the waveform when participants voluntarily attended the non-target repeated standard clicks, without *n*-back performance. The relative suppression of SN10 in *n*-back tasks, particularly the 3-back is consistent with the notion that load suppresses attention during the SN10 time range. *Credit:* Adapted from Sörqvist et al. (2012); *n* = 35. Reprinted by permission of MIT Press Journals.

To investigate the effect of concurrent visual-verbal memory load and WMC on the subcortical processing of sound, Sörqvist et al. (2012) employed an *n*-back task with young adults who had normal hearing: An *n*-back memory task was accompanied by large numbers of task-irrelevant clicks. Those clicks elicited ABRs. Visual letters appeared one-at-a-time and participants attempted to press a button when a letter was the same as that *n* letters ago. *n*-back tasks with higher *n*s thus meant higher concurrent memory loads. Performance was indeed poorer with higher memory load (1-back = 2-back < 3-back), while the Wave V of the ABR decreased (1-back < 2-back = 3-back). Also identifiable in the ABR data of Figure 12, was a SN10 negativity that also decreased (1-back = 2-back > 3-back).

This influence of simultaneous visual-verbal memory load shows that systems of WM affected those systems that influence the generators of wave V. Those generators are within or near the rostral brainstem. WM systems also affected the generators of SN10, which have cortical contributions (Parkkonen et al., 2009). Though Sörqvist et al. (2012) revealed that this SN10 more closely mirrored behavior, there was a functionally relevant influence of memory load on wave V (4–8 ms). When that influence exceeded a certain threshold, higher memory loads accordingly affected the subsequent SN10 (10.5–12 ms), possibly via the ascending auditory system. Either common processes affect the generation of SN10 and the brain mechanisms supporting *n*-back performance or SN10 generation and *n*-back share common processes. As the effect on wave V was prolonged, overlapping Waves III (4 ms) and IV (5 ms), the effect of load is not focal to the lateral lemniscus terminating in the IC that determines the peak of wave V (Møller et al., 1994). Rather, the effect could be mediated by the IC itself generating a slower longer-lasting waveform overlapping wave III and IV, possibly extending to influence the SN10. There was also further compelling evidence for the functional relation of memory load on this longer lasting aspect of wave V generation. This evidence stemmed from data on complex span tasks including the OSPAN (Turner and Engle, 1989; Beaman, 2004). All participants in the *n*-back also separately completed these complex span tasks to determine WMC. WMC is the maximum number of items that can be stored during the processing of the task and recalled correctly after near flawless performance of that task. A task accuracy criterion ensures that there is no trade-off between the task and memory storage. While performance on the task can be subject to momentary performance aberrations, WMC is a cognitive trait of a person: a long-term measure of that person's cognitive competence.

Sörqvist et al. (2012) correlated WMC from complex span tasks with deflections of the ABR to clicks that were measured during the separate *n*-back task. The concept was to determine if WMC predicted ABR generation under the different memory demands of different *n*-back tasks. Only with the higher memory demands of the 3-back, did individuals' WMC predict Wave V amplitude. On this 3-back, yet not the 1-back and 2-back, the higher the individual's WMC, the lower the amplitude of Wave V.

Sörqvist et al. (2012) postulate that the prefrontal lobe is at the apex of an attentional network supporting WM and the top-down suppression of the processing of incoming sound stimuli—stimuli that receive preliminary processing by the brainstem. In accordance with an assumption of top-down cholinergic control, Sörqvist et al. (2012) hypothesize that prefrontal projections to the cortical cholinergic system, reliant on the neurotransmitter acetylcholine, can suppress to-be-ignored sound (Sarter et al., 2005). Corticofugal connections of the descending auditory system could mediate that suppression. Accordingly, Wave V and SN10 is so-affected by WM load. This load-dependent reduction supports a limited prefrontal capacity assumption: Engaging a capacity-limited prefrontal control with a (visually presented memory) load diverts predictive selectivity away from processing the to-be-ignored clicks. The processing of clicks is thus suppressed at the level of the rostral brainstem. In accordance with an assumption of predictive selection by an early filter, this suppression may be particularly effective when the to-be-ignored sounds are highly predictable. Sörqvist et al.'s (2012) oddball sequences contained largely repetitions of the same click sound. As we already postulated, WM for such recent acoustical context may be crucial to perceptual compensation (Watkins and Raimond, 2013) that attenuates the effects of reverberation on speech perception. Sörqvist et al.'s (2012) evidence shows that there is an interplay between an individual's WMC and the corticofugal suppression of the generation of Wave V. As this interplay between an individual's WMC and this corticofugal suppression is arguably cholinergic, we term that interplay the cholinergic working memory assumption. This interplay is particularly apparent when all sound is to-be-ignored and the primary task requires a higher memory load.

Germane to the mechanisms of this effect of WM on brainstem processing is a recent investigation that demonstrated an age-related decline in WMC in audiometrically normal adults. This investigation measured WMC with a complex span task (Füllgrabe et al., 2015). There were two audiometrically matched groups of such individuals, an elder group, aged 60–79 years, and a younger group, aged 18–27 years. The decline in WMC was associated with a decline in speech-in-noise performance. The association of speech-in-noise performance with WMC at first appeared to be entirely mediated by aging: Statistically controlling for age eliminated this correlation (Füllgrabe et al., 2015). However, a larger-scale investigation of audiometrically normal individuals (Füllgrabe and Rosen, 2016) revealed that this association withstood statistical control for age. On balance, reconciling the data of Füllgrabe et al. (2015) with those of Füllgrabe and Rosen (2016), there is an influence of an age-related decline of WMC, which is associated with a decline in speech-in-noise performance. This influence is stronger in more elderly individuals. Age-related declines in WMC were not the only factor, as individual differences in WMC within a limited age range also predicted speech-in-noise performance. The association was moderate and significant separately in the elder groups aged 40–59 years, 60–69 years, and 70–91 years, yet weak and non-significant in individuals aged 18–39 years (Füllgrabe and Rosen, 2016). Füllgrabe et al. (2015) further revealed that better sensitivity to TFS information was also associated with improved speech-in-noise performance. This TFS processing was also subject to age-related decline. However, other individual differences between participants, which varied within but not between age groups, also affected that TFS processing: Performance on some cognitive tests exhibited moderate-to-strong positive correlations of better performance with improved TFS sensitivity. These cognitive tests were forward digit span, backward digit span, as well as sub-tests of the Test of Everyday Attention (TEA): trail making, block design, map search, and elevator counting with reversal. Overall, better scores on TEA also correlated positively with improved TFS sensitivity. These correlations were moderate, but remained significant after partialling out the effect of age. By contrast, Füllgrabe et al. (2015) revealed no significant association between measures of TFS perception and WMC, cohering with the notion that cortical cholinergic mechanisms are not the only mechanisms modulating the subcortical processing of TFS. Performance on some cognitive tests correlated positively with TFS sensitivity after partialling out the effect of age. Some mechanisms for processing TFS are thus resistant to the effects of aging. We postulate that, even in those with normal hearing, there is a distinct age-related decline in the cortical cholinergic system impacting the influence of the prefrontal lobe on brainstem processing. In turn, that decline affects the representation of TFS by the rostral brainstem, thus determining speech-in-noise performance. Potential cholinergic mechanisms for such an age-related decline could include the age-related damage of post-synaptic muscarinic acetylcholine receptors. Positron Emission Tomography has revealed an age-related reduction in the binding of such receptors in the neocortex and thalamus (Zubieta et al., 2001).

In accordance with a cholinergic top-down control assumption, the extent of age-related decline of such cholinergic mechanisms, we postulate, determines how top-down expectancies can corticofugally modulate the subcortical representation of speech TFS information. This TFS information is crucial to the processing of speech-in-noise retained by elderly individuals with age-normal hearing (mild-to-moderate loss) and arguably represented at the level of the rostral brainstem (Fujihira and Shiraishi, 2015). Indeed, populations of neuronal elements simultaneously firing in the rostral brainstem represent in phase-locked manner such information up to 1500 Hz (Aiken and Picton, 2008); higher frequencies arguably relying on a rate-place code (Rhode and Greenberg, 1994; Skoe and Kraus, 2010) via tonotopy without phase-locking in the IC (Harris et al., 1997). The data of Füllgrabe et al. (2015) also point toward additional mechanisms for processing TFS, which are associated with speech-in-noise performance yet are relatively resistant to the influence of age-related decline. These mechanisms are neurocognitive processes that are unaffected by aging yet contribute to performance on several cognitive tasks. By contrast, Füllgrabe et al. (2015) revealed no significant association between measures of TFS perception and WMC, cohering with the notion that cortical cholinergic mechanisms are not the only mechanisms modulating the subcortical processing of TFS. Such mechanisms for representing TFS could more critically implicate inhibitory GABA in the inferior colliculus as could also be affected by aging (Caspary et al., 2008; Anderson et al., 2011) though without directly affecting WM. An alternative tenable hypothesis could also concern age-related declines in excitatory serotonergic neurotransmission in the ascending central auditory system (Tadros et al., 2007).

### Section summary

Increases in concurrent cognitive memory load affect the brainstem processing of sound in a manner that attempts to shut-down that processing by the auditory brainstem. That shut-down conforms with the notion that corticopetal-corticofugal loops of the early filter operate according to the assumptions of predictive selection and a prefrontal capacity-limitation. This attempt to shut-down brainstem processing is thus top-down and constrained by WMC. Acccordingly, under conditions of high concurrent memory load, those with higher WMC show a reduced wave V. The facet of WMC that declines with age, alongside the age-related decline in TFS processing, affects speech-in-noise performance. These influences of WMC support a cholinergic working memory hypothesis: People with better WMC for the storage and processing of acoustical context, as measured by complex span tasks, possess better prefrontal control of corticopetal-corticofugal loops via the cortical cholinergic system.

We postulate a cholinergic stance of the cognitive load on perception hypothesis, that, even in those with normal hearing, there is an age-related decline in the cortical cholinergic system. This cortical cholinergic decline impacts the influence of the prefrontal cortex on brainstem processing and, in turn, the sensory-cognitive representation of TFS determining speech-in-noise performance. There are likely other age-influenced mechanisms affecting TFS processing that are not influenced by age-related declines in WM, such as those implicating collicular GABA.

Potential cholinergic mechanisms for an age-related decline affecting WM and speech-in-noise performance include the age-related damage of post-synaptic muscarinic acetylcholine receptors. Here we have seen WMC constrains the influence of cognitive load on the subcortical processing of sound, as could be related to the processing of speech in noise. We now turn to intriguing parallels concerning the influence of sensory load on the behavioral effects of processing to-be-ignored sound in auditory distraction paradigms.

## Sensory load and auditory distraction

The disruptive effects of auditory distraction upon WM have been extensively investigated in a serial recall paradigm (Jones, 1993). To-be-remembered items are presented one-at-a-time and a to-be-ignored sequence of sounds is presented alongside those items and/or during a distraction-filled retention interval. Hughes et al. (2013) investigated two auditory distraction effects in this paradigm. The first disruptive effect, produced by an occasional change-of-voice in the to-be-ignored sound, is termed a deviant effect. Hughes et al. termed the second disruptive effect the changing-state effect, variously known as the token set size effect (Campbell et al., 2002a). That is, a repeated sound *AAAAAAAAA*…is less disruptive that a changing sequence of multiple sound tokens *ABCDEABCDE*.…

**Figure 13:**
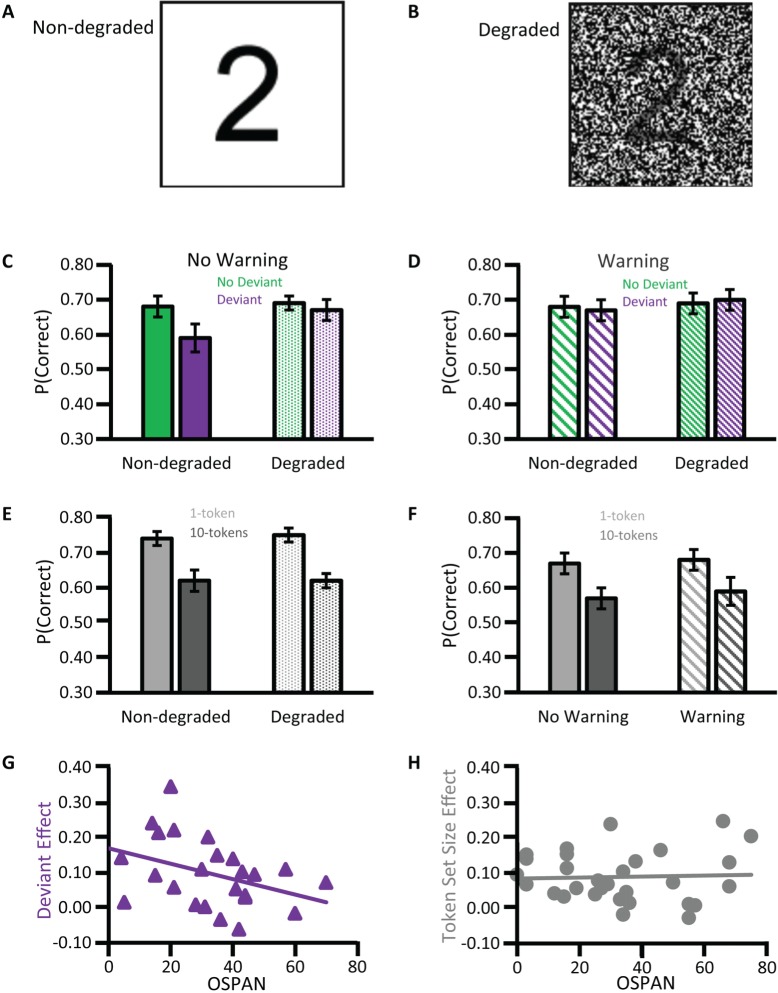
**Sensory load, foreknowledge, and working memory capacity affect the deviant effect, not the token set size effect**. Increasing sensory load by degrading to-be-remembered serial recall visual digit items **(A)** with Gaussian noise **(B)** reduces the deviant effect **(C)**, whereby an occasional change-in-voice of to-be-ignored speech disrupts serial recall performance. Foreknowledge of an imminent deviant eliminates this influence of sensory load and the deviant effect **(D)**; *n* = 24. A comparable influence of sensory load on the token set size effect or “changing-state effect” was not apparent **(E)**; *n* = 45, nor was there any modulation by foreknowledge of changing-state multi-token stimulation that was consistently more disruptive than a repeated speech token **(F)**; *n* = 31. Indeed, WMC as indexed by OSPAN correlated negatively with the deviant effect **(G)**; *n* = 24, yet not the token set size effect **(H)**; *n* = 31. *Credit:* Copyright © 2013 by the American Psychological Association. Adapted with permission from Hughes et al. (2013). The use of APA information does not imply endorsement by APA.

Hughes et al. found that increasing the sensory load, by taking visual to-remembered digits (Figure 13A) and degrading with Gaussian visual noise (Figure 13B), decreased the deviant effect (Figures 13A,C). Such an attenuation of the deviant effect with increases in sensory load thus resembled the attenuation of Wave V of the ABR by increases in *n*-back load (Sörqvist et al., 2012). Further, Hughes et al. also revealed that forewarning the participant of the presence of a deviant attenuated the deviant effect (Figure 13D). A viable interpretation is thus that a top-down expectancy led to a prefrontally coordinated and cholinergically mediated corticofugal influence on subcortical filtering. This interpretation thus assumes the early filter can operate according to a principle of foreknowlege: predictive selectivity that affects cholinergic top-down control of that filter. This filtering thus attenuated the disruptive influence of an expected rather than an unexpected change-in-voice. A further parallel with the ABR findings of Sörqvist et al. (2012) was compelling: The higher the OSPAN measure of WMC, the smaller the deviant effect (Figure 13G). This correlation has been replicated (Sörqvist, 2010) as further corroborated by meta-analysis (Sörqvist et al., 2013). Such a finding would be expected whereby a prefrontal cortex-coordinated WM system modulates the subcortical filtering of deviant to-be-ignored sound. The subcortical filtering would in turn attenuate that deviant's cortical processing and disruptive propensity.

By contrast to this deviant effect, the token set size effect went unmodulated by either sensory load (Figure 13E) or forewarning (Figure 13F) of the presence of a multi-token sequence. Indeed, that token set size effect went uncorrelated with WMC (Figure 13H). A top-down expectancy generated by a repeated token *AAAA*…may suffice for corticofugal influences on subcortical filtering to attenuate the cortical processing of that sound. A hypothetical top-down expectancy that attenuates the disruptive effects of a changing-state multi-token sequence varying in many attributes (rather than just the voice of speaker) seems to defy formulation. If such a top-down expectancy is formulated, any influence on distraction is swamped by other distraction-invoking mechanisms strongly influenced by token set size. For instance, the effects of increases in token set size on the supratemporal auditory cortex as indexed by releases in refractoriness of the supratemporal N1—that could be related to the form of auditory distraction termed the token set size effect (Campbell et al., 2003, 2005, 2007)—would accordingly go relatively unaffected by such corticofugal influences.

**Figure 14:**
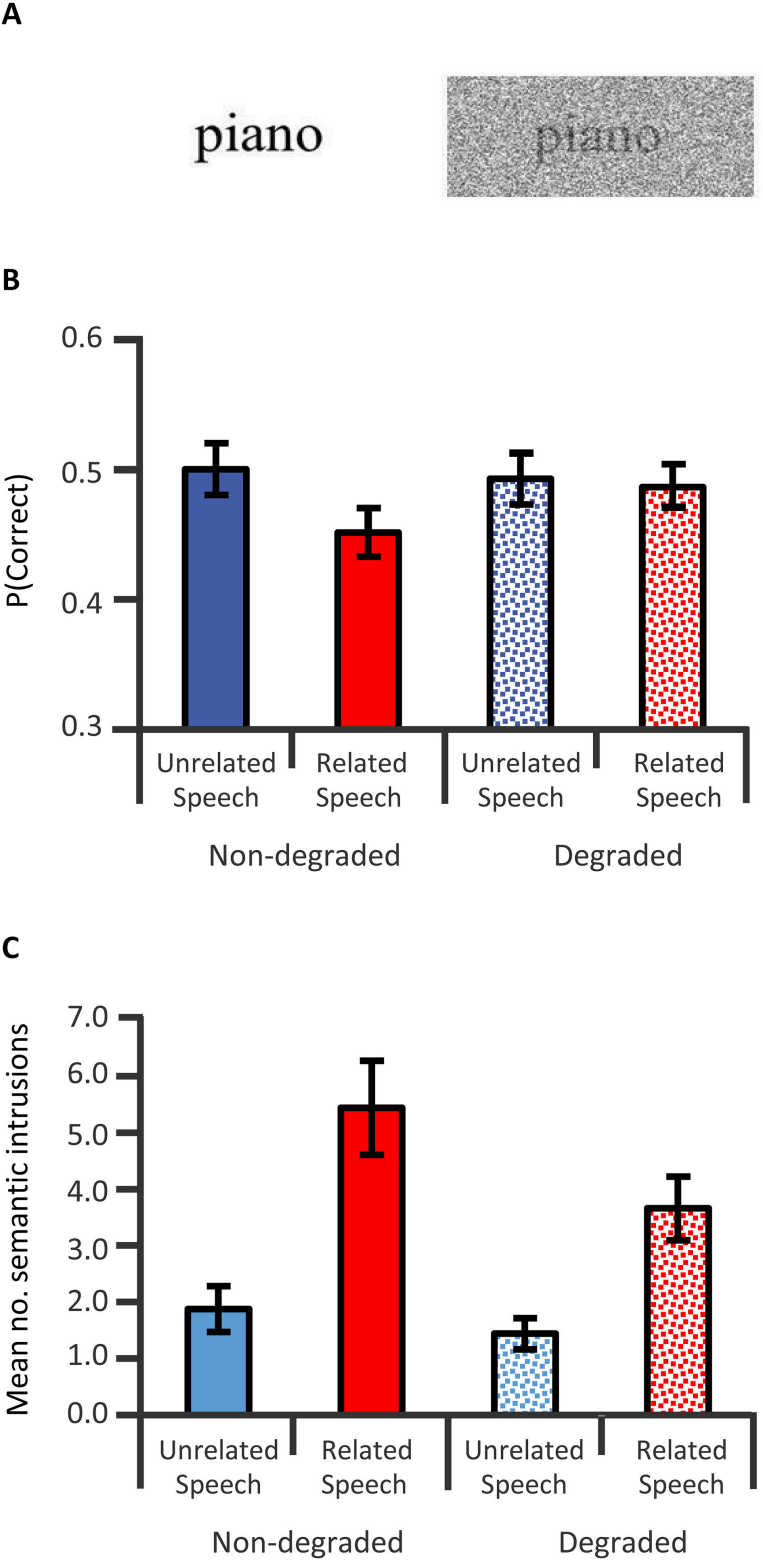
**Sensory load attenuates the between-sequence semantic similarity effect**. Increasing the sensory load **(A)** of the to-be-remembered target items (e.g., “chair, desk, wardrobe…”) reduced the influence of the meaning of to-be-ignored speech on WM performance **(B)**: Under such conditions of low sensory load, the semantically related to-be-ignored speech sound (e.g., “table, sofa, bookshelf…”) disrupted recall performance more than semantically unrelated speech (nurse, secretary, carpenter). This increase in load also reduced the influence of semantic relatedness on the number semantic intrusions into recall from the to-be-ignored speech **(C)**. Sensory load thus affects the influence of auditory meaning on cognitive processes, Marsh et al. (2015b: Exp.1); *n* = 32. *Credit:* Copyright © 2015 by the American Psychological Association. Adapted with permission from Marsh et al. (2015b). The use of APA information does not imply endorsement by APA.

Similar to the deviant effect, sensory load attenuates a semantically mediated phenomenon known as the between-sequence semantic similarity effect (Marsh et al., 2015b; Figure 14). Marsh et al. (2015b) presented to-be-remembered words visually and concurrently with to-be-ignored heard words. To-be-ignored words drawn from the same semantic category as the to-be-recalled words disrupt recall of those to-be-remembered words: Fewer to-be-remembered words were recalled with increases in the semantic relatedness of the to-be-remembered and to-be-ignored material (Figure 14B). Further, more of the to-be-ignored words erroneously intrude into recall (Figure 14C). Marsh et al. (2015b) revealed that degrading the visual word stimuli with Gaussian noise (Figure 14A), thereby increasing sensory load, modulated these effects of semantic relatedness (Figures 14B,C). We offer an interpretation of how sensory load reduces this between-sequence semantic similarity effect, consistent with how sensory load also reduces the deviant effect: A prefrontal cortex-coordinated WM system ultimately modulates the subcortical filtering of to-be-ignored sound promoting the cortical processing of semantically relevant auditory material. When a sensory load engages that prefrontal control, accordingly the top-down semantic expectancies (expectancies of a cognitive-linguistic nature) controlling the corticopetal-corticofugal loops no longer support the processing of semantically relevant auditory material. In turn, with a sensory load, semantically relevant material is less intrusive and less disruptive of the recall of the to-be-remembered material.

There is a sensory load of a slightly different sort, the presence of to-be-ignored background noise: speech-shaped noise accompanying to-be-recalled words (Marsh et al., 2015a). While not affecting the perception of to-be-attended items, such background noise rather can impair the semantic processing of the to-be-attended items. Theoretically, when the sensory load of this speech-shaped noise engages prefrontal control, top-down semantic expectancies, corticofugally controlling the corticopetal-corticofugal loops, no longer support the processing of the semantically relevant auditory material. Listening in noise thus recruits WM resources that would otherwise be used for elaborate semantic processing of spoken words (McCoy et al., 2005; Kjellberg et al., 2008). Noise disrupts that elaborate semantic processing. Therefore, the sensory load of background noise impairs the understanding of heard speech. Here we thus postulate that sensory load by to-be-ignored background speech sound engages prefrontal control of the cortical cholinergic system. Accordingly, semantic expectancies cannot corticofugally control the corticopetal-corticofugal loops that tune the subcortical representation of attended speech to semantically likely candidate utterances.

Marsh et al. (2015a) demonstrated that semantic processing is disrupted by noise, in carefully calibrated circumstances in which the perception of speech in noise is relatively unimpaired. However, top-down semantic expectancies have been shown to support the contextual repair of degraded sensory information thereby improving speech perception (Shahin and Miller, 2009; Shahin et al., 2012). Accordingly, the engagement of prefrontal control by to-be-ignored speech could adversely affect the influence of semantic expectancies on the perception of speech in noise.

### Section summary

Here we draw parallels between the WMC constrained influence of cognitive memory load on the subcortical processing of sound and the influence of sensory load on forms of auditory distraction. In both cases, increases in load had effects that were constrained by WMC. Though some forms of auditory distraction go unaffected, we postulate that (semanto-syntactic) foreknowledge affects the top-down corticofugal influences of the cortical cholinergic system that is influenced by WM. Those influences affect subcortical processing alongside the processing of auditory deviance and auditory meaning, thus influencing the perception and understanding of speech in noise. These findings from distraction and speech-in-noise findings have motivated the assumptions of the new early filter model to which the discussion now turns.

## The new early filter model

**Figure 15:**
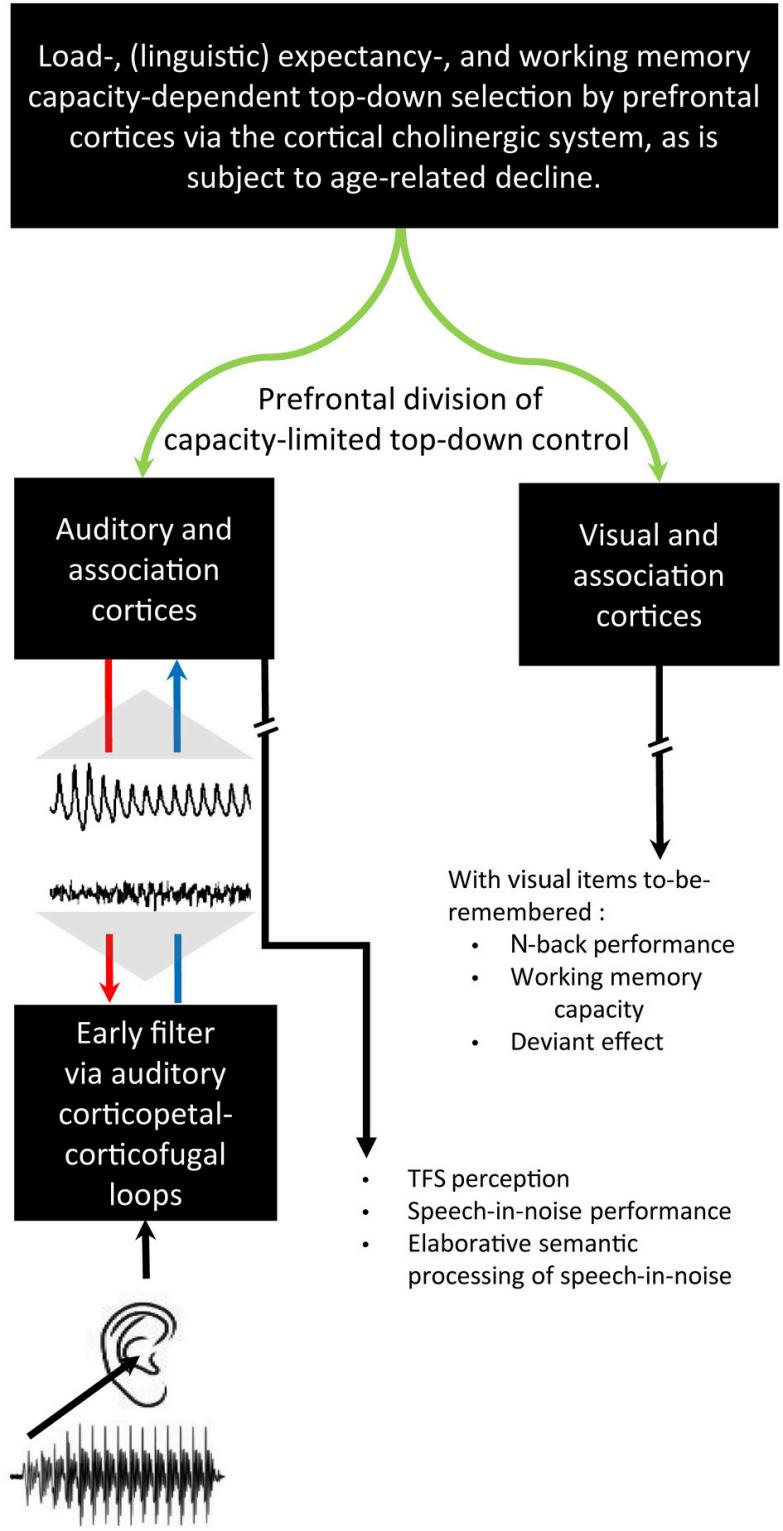
**The new early filter model**. *Credit:* Waveforms reprinted from Fujihira and Shiraishi (2015). Copyright © 2015, with permission from Elsevier; *n* = 30.

The new early filter model is depicted in Figure 15. Corticofugally controlled corticopetal-corticofugal loops serve as an early filter increasing the signal-to-noise ratio at the cortex, operating early by egocentric selection (Suga et al., 2000). This selection serves to enhance the predicted signals and suppress unattended predicted noise. For instance, as Figure 2 illustrates, one corticopetal-corticofugal loop includes corticopetal connections ascending from the right IC up to the right auditory cortex via the right medial geniculate body. This loop also includes corticofugal connections descending from the right auditory cortex to the right IC via the right medial geniculate body. Such a loop receives not only information from loops lower in the central auditory system, but also controls those lower loops. This loop also sends information upward and is under the control of higher loops. The representation of the auditory speech signal at the level of the rostral brainstem is well-specified as phase-locked synchronous activity up to 1500 Hz. The fidelity of that representation of TFS information of the to-be-attended auditory signal, supported by a phase-locking over a broad region of inferior colliculus (Harris et al., 1997), arguably limits the processing of speech-in-noise, affecting in turn word recognition by the cortex (Fujihira and Shiraishi, 2015).

The new early filter model revives Broadbent's (1958) influential *early filter assumption*: There is a capacity limitation on how the human mind processes information that selects information early on for further processing. A psychological theoretical difference is that the new early filter model assumes that prior contextual information, which a working memory network stores and processes, can determine an attentional expectancy.

The prefrontal cortex is not only an aspect of that working memory network (Gisselgård et al., 2004; Campbell, 2005) but also an aspect of the anterior attentional system (Sarter et al., 2005). Attentional requirements and an attentional expectancy derived from prior context affect the prefrontal control of the cholinergic basal forebrain that in turn can cholinergically top-down control the organization of the primary auditory cortex (Kilgard and Merzenich, 1998). This we term the *cholinergic top-down control assumption*.

The key departure from Broadbent (1958) is that the early filter of corticofugal-corticopetal loops is by default wide open, such that, when stimulation in unpredictable, late selection may be more influential than early selection on cognitive performance. However, when (linguistic) expectancy predicts the to-be-attended stimulation, then that early filter becomes more selective. This we term the *predictive selection assumption*.

This predictive selectivity can improve TFS sensitivity and speech-in-noise perception. Also this predictive selectivity renders the cABR to selected information more faithful to the (linguistically) predictable stimulus: neural entrainment. We postulate predictive selectivity via corticofugal-corticopetal loops not only affects the perception of speech in noise, but also affects the comprehension of speech in noise. Prefrontal control is assumed to be capacity-limited. This we term the *prefrontal capacity limitation* assumption.

Accordingly, a sensory or a cognitive load on that prefrontal control, diverts predictive selectivity away from other stimuli. There is thus a cognitive load-dependent reduction of the wave V evoked by to-be-ignored clicks (Sörqvist et al., 2012) due to a diversion of prefrontal control toward the control of information processing within visual and association cortices.

Combining the predictive selectivity assumption and the prefrontal capacity limitation assumption also accounts for several semantic phenomena. The sensory load of meaningless speech-shaped noise disrupts the elaborative semantic processing of the to-be-attended speech in that acoustical noise (Marsh et al., 2015a). This noise diverts prefrontal control toward processing the sensory load of acoustical noise in visual and association cortices: There is a diversion of prefrontal control away from the storage and processing required for using preceding sound to predict what the semantically likely candidate utterances are. Similarly, the sensory load of visual noise diverts prefrontal control away from the cognitive processes required for the encoding of visual items into memory (Marsh et al., 2015b). That prefrontal control is diverted toward processing the sensory load of the visual noise. This visual sensory load also diverts prefrontal control away from the semantic processing of to-be-ignored sound and to-be-remembered visual items, thus abolishing the between-sequence semantic similarity effect (Marsh et al., 2015b). In turn, that visual sensory load diverts prefrontal control away from the involuntary attentional processing of a to-be-ignored change of voice, thus decreasing the deviant effect (Hughes et al., 2013).

People with better WMC for the storage and processing of acoustical context posess better prefrontal control of corticopetal-corticofugal loops via the cortical cholinergic system. This we term the *cholinergic working memory* assumption. These individuals thus have enhanced load-dependent reductions of the wave V elicited by to-be-ignored clicks (Sörqvist et al., 2012). Further, combining the cholinergic working memory and prefrontal limited capacity assumptions with the predictive selectivity assumption offers explanatory value. This combination of assumptions accounts for how higher WMC-individuals show better resistance to the deviant effect (Hughes et al., 2013) and, in a different manner, better speech-in-noise performance (Füllgrabe and Rosen, 2016). We turn first to the deviant effect.

A person's WMC affects the prefrontal control of the early filter's predictive selectivity via the influence of cholinergic projections of basal forebrain on corticopetal-corticofugal loops. With higher-WMC participants, who have better prefrontal control of corticopetal-corticofugal loops, there is top-down cholinergic control that tunes predictive selectivity well. That better tuning prevents extensive processing of the deviant change of voice in the to-be-ignored sound, thus reducing the deviant effect (Hughes et al., 2013). That deviance would otherwise capture prefrontal control away from the visual and association cortices, which support the encoding of the to-be-remembered items into working memory. The notion of corticofugal influences of visual attention on auditory deviance processing agrees with data concerning the auditory mismatch negativity (Campbell, 2015). Prefrontal influences of visual attention on subcortical auditory filtering by corticopetal-corticofugal loops could also, in turn, permit visual attention to influence the cortically generated auditory supratemporal mismatch negativity (Erlbeck et al., 2014; Campbell, 2015). The deviant effect, could be related, at least in part, to the auditory deviance processing that this mismatch negativity indexes. Indeed, there are stronger cholinergic influences on the auditory mismatch negativity in young individuals (Pekkonen et al., 2001) than in elder adults (Pekkonen et al., 2005). Pekkonen et al.'s findings thus arguably cohere well with the cholinergic working memory assumption: Elder participants also have reduced complex span performance (Bopp and Verhaeghen, 2005) such that the cortical cholinergic system no longer strongly influences deviance processing in those older adults (Zubieta et al., 2001; Pekkonen et al., 2005). Low-WMC participants, who arguably have less effective cortical cholinergic systems, show stronger deviant effects (Hughes et al., 2013). Foreknowledge of an imminent deviant similarly attenuates the deviant effect. This foreknowledge provides WM with a top-down context that the prefrontal anterior attentional system uses to cholinergically improve that predictive selectivity (Hughes et al., 2013). In turn, this effect of foreknowledge on predictive selectivity excludes the processing of deviance via an early filter through the control of corticofugal-corticopetal loops. Such contextual influences of foreknowlege is assumed to play a role in how top-down (semanto-syntactic) expectancies can improve speech-in-noise performance. This we term the *foreknowledge predictive selectivity* assumption.

Having discussed the implications for understanding the deviant effect of combining the cholinergic working memory and prefrontal limited capacity assumptions with the predictive selection assumption, we turn now to speech-in-noise perception itself. The processing and storage of acoustical context to promote predictive selectivity is better in higher-WMC participants. These higher-WMC participants thus have better speech-in-noise perception. While this correlation was significant for participants aged 18–91 years, listeners aged 40–91 years caused this association between WMC and speech-in-noise perception to be significant (see Füllgrabe and Rosen, 2016). What the cholinergic facet of the cholinergic working memory assumption contributes to this explanation is a biological mechanism. This mechanism is assumed to be that by which the age-related decline in WMC predicts declines in speech-in-noise performance. Cholinergic decline (Zubieta et al., 2001) thus led to a decline in the influence of the prefrontally controlled cholinergic basal forebrain. This decline would not only affect the anterior attentional system, including the prefrontal cortices that are part of a working memory network (Campbell, 2005) thus affecting WMC for visually presented material. That decline would also affect the cholinergic basal forebrain's control of the auditory cortex (Kilgard and Merzenich, 1998) in turn adversely affecting speech-in-noise perception. A cholinergic stance of Schneider and Pichora-Fuller's (2000) cognitive load on perception hypothesis would thus predict that a cognitive aging of the cortical cholinergic system drives a decline in sensory processing.

### Section summary

The new early filter model assumes prefrontal cortex controls top-down expectancy via the cortical cholinergic system thus influencing sensory and association cortices. In turn, the cholinergic basal forebrain indirectly influences corticopetal-corticofugal loops by corticofugal descending connections, as is termed cholinergic top-down control. Those corticopetal-corticofugal loops serve as an early filter, acting upon the level of the rostral brainstem. This filter operates according to the assumption of predictive selection such that expectancies on the basis of preceding stimulus context, linguistic context, or foreknowledge affects TFS perception and speech perception in noise/reverberation. Combining the predictive selection assumption with that of a prefrontal capacity limitation has explanatory advantages. This combination explains how diversions of prefrontal control lead to load-dependent reductions of wave V, alongside several semantic phenomena. One such phenomenon is how meaningless noise disrupts the semantic elaborative processing of speech in that noise. The cholinergic working memory assumption that complex WMC affects the early filter via the cholinergic basal forebrain's control of corticopetal-corticofugal loops has further explanatory value. The addition of this assumption explains how WMC influences both load-dependent reductions in wave V and speech-in-noise performance.

## Explanatory limits of the early filter

Having discussed the explanatory value, we turn to the explanatory limits of the new early filter model with respect to auditory distraction and speech in noise. The form of auditory distraction known as the changing-state or token set size effect that, in theory, relates to the refractoriness of the generation of the supratemporal N1 (Campbell et al., 2003, 2005, 2007) is arguably unrelated to the cortical cholinergic system. Expectancy or sensory load thus does not affect that form of distraction (Hughes et al., 2013). Though there may be cholinergic influences on the latency of auditory N1 generation, the cholinergic antagonist scopolamine does not affect the refractoriness of the generation of the M100 magnetic counterpart of the supratemporal N1 (Pekkonen et al., 2005). Further, there is support for an influence of a separate, at least partially GABAergic influence on the latency of supratemporal M100 generation (Gandal et al., 2010). The MEGAPRESS technique—which is insensitive to acetylcholine—revealed high GABA+ macromolecule measurements in an auditory region of interest extending from middle temporal regions to superior temporal gyrus (Gaetz et al., 2014). This finding arguably indicates that Gandal et al.'s modulation of M100 generation is in part GABAergic. The token set size effect that could be related to refractoriness of N1 generation (Campbell et al., 2003, 2005, 2007) and M100 generation is, however, not necessarily completely unrelated to speech-in-noise performance: Noise can produce an auditory distraction effect influencing the cortical retention of linguistic information in turn limiting the perception and understanding of speech in noise. This noise produces a stronger auditory distraction effect with fluctuating changing-state or multi-token noise than steady-state noise.

The reverberatory adverse conditions of interfering high intensity speech in a restaurant, with a vaulted non-absorptive ceiling, present a high sensory load under which to attempt to listen to the attended speech. In such circumstances, an early filter arguably attempts to top-down attenuate the ABR (Sörqvist et al., 2012). This filter attempts to close-down the processing of auditory noise at the cost of closing-down the processing of the auditory signal. However, those conditions also present a token set size effect that is resistant to such top-down effects. Alternatively, those conditions could even produce a stronger form of auditory distraction under conditions of high cognitive load (Gisselgård et al., 2003, 2004; Valtonen et al., 2003; Campbell, 2005; Petersson et al., 2006). This token set size effect can affect the perception of and memory for lipread material, when that perception benefits from the retention of contextual information (Campbell, 2000; Campbell et al., 2002b): For some individuals directional microphone(s) might sufficiently reduce sensory load for the perception and understanding of speech. Others might attain more effective communication in such adverse conditions by lip-reading, closing-down their hearing by switching-off hearing assistive devices, or even by covering one's ears.

## Open questions and caveats for future research

The new early filter model assumes that WMC constrains processing of sound at the rostral brainstem according to top-down expectancies. Convergent evidence supports this assumption from effects of load and WMC on ABRs, alongside different forms of auditory distraction.

The proposed mechanism for controlling this filter is a prefrontally coordinated network that supports WMC and controls the cholinergic basal forebrain. This cholinergic basal forebrain, in turn, can modulate corticopetal-corticofugal loops controlling the subcortical early filtering of auditory information. We postulate a representation of the preceding context, which a WM network—including the prefrontal cortices—maintains and manipulates. The processing of that representation permits top-down prediction that selects the perceptual representation of the current utterance supporting the auditory perception of speech. Accordingly, that WM interacts (cholingerically) with an early stage of processing in the brainstem to support that predictive selection by the early filter.

This filter is wide open when top-down expectancies defy formulation, such as during highly variable meaningless sequences of speech noise information, (e.g., Campbell et al., 2003, 2005) *jus, käs, tam, nev, poi, tam, jus, käs*…This notion is thus reconcilable with evidence previously martialed in favor of attenuation or late selection models of auditory attention. Yet it is viable that top-down expectancies and cortical modulations responsive to the dynamics of meaningful speech control corticofugal connections mediate subcortical neural entrainment. This conjecture leads to an open empirical question for cABR investigations: Is there a syntactically or semantically mediated form of subcortical neural entrainment? A caveat for cABR investigations to reveal a compelling semanto-syntactic influence on such neural entrainment is that the signal-to-noise ratio of the cABR needs to be high. To do so is a methodological challenge with ordinary EEG equipment, as requires epoching EEG to the onsets of thousands of sounds (e.g., Campbell et al., 2012). Comparing neural entrainment using sequences of semantically or grammatically related word sounds rather than unrelated pairs of word sounds could thus be more practical than using large numbers of sentences. A further caveat is, for that entrainment to be established as subcortical, the cABRs measured need to be unconfounded by cortical contributions of the SN10 (Parkkonen et al., 2009). It is thus necessary to digitally filter cABR recordings in a way that substantially removes the SN10 to click ABRs from the same session. This filtering should not remove Wave V of the ABR.

Open empirical questions of practical and theoretical importance arise for which the new early filter model offers a framework for making predictions. The model predicts, as already established, that (younger) high-WMC participants would be better at hearing words within noise. Yet those high-WMC participants should also show a decreased between-sequence semantic similarity effect when those words serve as the to-be-ignored speech. Open research questions also relate to treatments for hearing loss such as neuropharmacological approaches and WM training. The cholinergic stance of the cognitive load on perception hypothesis concerns an age-related decline in the cortical cholinergic system. This hypothesis would predict that, for aging individuals exhibiting post-synaptic muscarinic acetylcholine receptors damage, use of acetlycholinesterase inhibitors could improve WM function for complex span tasks. In turn, this pharmacological treatment would also improve TFS perception alongside the perception and comprehension of speech in noise. WM training may have similar effects. Schneider and Pichora-Fuller's (2000) sensory deprivation hypothesis, assuming sensory decline drives chronic cognitive decline, should be borne in mind. Even in audiometrically normal individuals, those persons could have a hidden peripheral loss. Accordingly, that loss would result in a sensory decline that may drive damage to post-synaptic muscarinic acetylcholine receptors thus producing cognitive decline. As such, in experiments testing this cholinergic stance of the cognitive load on perception hypothesis, in selecting participants of all ages, screening should not only use audiograms but also use ABR measures of hidden loss, such as the ratio of wave I to wave V (Schaette and McAlpine, 2011). We offer a caveat for the interpretation of evidence from pharmacological treatments seeming to support a cholinergic stance of the cognitive load on perception hypothesis. Those treatments may effect variables such as attributes of cABRs, TFS sensitivity, WMC, or the perception and comprehension of speech in noise. The caveat is that the individuals undergoing the intervention should neither exhibit audiometric nor hidden loss.

Open questions also concern the relation of peripheral sensorineural hearing loss to a compensatory dedication of cognitive resources to the perception and understanding of speech under adverse conditions. Further open questions concern how such a compensation relates to the age-related decline of these systems of neurotransmission alongside an accelerated decline in cognitive faculties including WM.

## Author contributions

Both JM and TC made substantial contributions to the concept and interpretation in drafting the manuscript, approved the submitted materials, and have agreed to be accountable for all aspects of the work in ensuring that questions related to the accuracy or integrity of any part of the work are appropriately investigated and resolved.

## Funding

The writing of this article was supported by a grant from the Swedish Research Council (2015-01116) awarded to Patrik Sörqvist and to JM.

### Conflict of interest statement

The authors declare that the research was conducted in the absence of any commercial or financial relationships that could be construed as a potential conflict of interest.

## Footnotes

^1^To obtain this subsequent SN10 (Davis and Hirsh, 1979), a wide input bandpass, such as 40–3000 Hz, is required in EEG measurements. An SN10 in the click ABRs was apparent (Figure 1A; Campbell et al., 2012) with a low cutoff within the recommended range for cABRs of 30–100 Hz (Skoe and Kraus, 2010). While the origins of the SN10 include the inferior colliculus in the brainstem, the data of Parkkonen et al. (2009) suggested that there is a contribution of contralateral cortical sources of the SN10 and thus warrant some words of caution. The recommended approach (Skoe and Kraus, 2010) is to record with a low cutoff of 1 Hz and then to filter digitally offline. Recording click ABRs on the same day as the cABRs is also conventional. To prevent the strong, possibly cortical, contributions of SN10 to cABRs, a recommendation for cABRs is thus a digital filter that substantially removes the SN10 to click ABRs from the same session. That filter should not remove wave V of the ABR.

^2^Declines in the processing of TFS are not necessarily in the brain or auditory nerves, but rather may be consequences of decline in the auditory periphery. TFS sensitivity does indeed decline with age even in the absence of peripheral hearing loss as measured by the audiogram (Füllgrabe et al., 2015). The loss of auditory nerve fibers or their synapses with hair cells, cochlea synaptopathy, can be age-related or noise-induced (Kujawa and Liberman, 2015). Plack et al. (2014) offer an account of how such noise-induced hearing loss can be audiometrically “hidden” (Schaette and McAlpine, 2011) yet might have consequences for temporal processing in the central auditory system, as indexed by FFR. Plack et al. present supportive preliminary data of how audiometrically normal young adults, who had a history of loud noise exposure, showed FFRs less faithful to a 3.1 kHz tone. It is as viable that age-related cochlear synaptopathy might drive a comparable age-related decline in temporal processing by the central auditory system, as reflected by Marmel et al.'s (2013) FFRs.
