# Supplementary material for: Processing Complex Sounds Passing through the Rostral Brainstem: The New Early Filter Model
Source: Front Neurosci. 2016 May 10;10:136. doi: 10.3389/fnins.2016.00136 (PMC4861936; doi:10.3389/fnins.2016.00136)
Supplement: Supplementary file 1 [file Image1.PDF]

Supplementary Material

Processing complex sounds passing through the rostral brainstem: The new early filter model

John E. Marsh*, Tom Campbell

*** Correspondence: Corresponding Author: jemarsh@uclan.ac.uk**

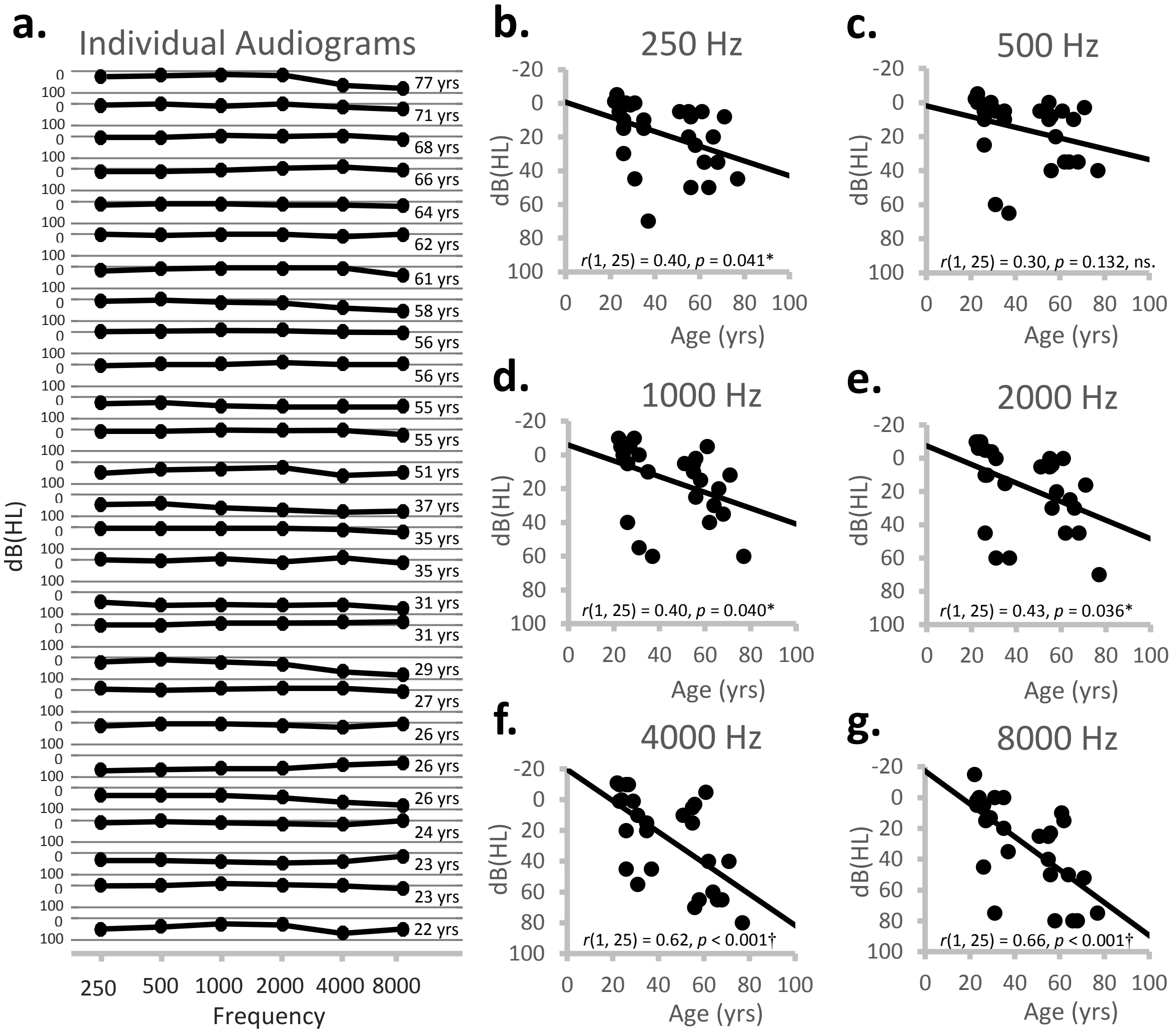

Supplementary Figure 1. Individual best-ear Pure Tone Audiograms as a function of ascending age (a), adapted with permission from Marmel *et al.* (2013). For each frequency separately, scatterplots (b-g) reveal positive correlations of hearing loss with age that were moderate at low frequencies (b, d, e; denoted by *) and slightly stronger at higher frequencies (f, g; denoted by †). These correlations confirm the tendency to sloping loss is more pronounced in older individuals, as illustrated in (a). Noteworthy is that at 500 Hz (c), the weak correlation is not significant. Frequency difference limens and FFR were assessed using stimuli close to this 500 Hz; n = 27.
